# Protective holobiome promotes strawberry tolerance of biotic stresses

**DOI:** 10.1007/s44154-026-00294-5

**Published:** 2026-03-24

**Authors:** Jin-Soo Son, Su Yeon Lee, Mee Kyung Sang, Francesco Spinelli, Choong-Min Ryu

**Affiliations:** 1 Molecular Phytobacteriology Laboratory, Infectious Disease Research Center, KRIBB, Daejeon, 34141 South Korea; 2 Division of Agricultural Microbiology, National Institute of Agricultural Science, Rural Development Administration, Wanju, 55365 South Korea; 3 Department of Agricultural and Food Sciences, University of Bologna, 40126 Bologna, Italy; 4 Department of Biosystems and Bioengineering, KRIBB School, University of Science and Technology, Daejeon, South Korea

**Keywords:** Biological control, Biotic stress, Holobiome, ISR, PGPR, Strawberry

## Abstract

The commercial cultivation of strawberry (*Fragaria × ananassa*) is increasingly challenged by biotic stresses such as plant pathogens and insect pests, while climate change exacerbates abiotic stresses. Reliance on chemical fumigants and broad-spectrum pesticides presents risks to human health, environmental quality, and microbial diversity. The strawberry holobiome, defined as the integrated community of plant-associated microorganisms that inhabit the rhizosphere, phyllosphere, endosphere, and fruit surface, is emerging as a key determinant of plant health and productivity. Recent metagenomic and metabolomic studies have identified cultivar-specific microbial consortia that suppress plant disease, enhance stress tolerance via induced systemic resistance, and modulate fruit quality. The engineering of synthetic microbial communities (SynComs) offers a targeted approach to microbiome augmentation, but the lack of high-resolution functional data hinders the development of effective SynComs, especially in hydroponic and substrate culture systems. This review synthesizes recent advances in holobiome profiling, evaluates microbial biocontrol strategies against major pathogens, and outlines future directions, including AI (artificial intelligence)-driven community design, integrated multi-omics analysis, and microbiome-assisted breeding. Addressing these gaps will enable precision management of the strawberry microbiome to sustain yield, quality, and resilience under dynamic environmental conditions.

## Introduction

Strawberry (*Fragaria × ananassa*) is one of the most economically important and nutritionally valuable berry crops cultivated worldwide (Simpson 2018; Zhang et al. 2010). The modern cultivated strawberry is a relatively recent domesticate that originates from a natural hybridization between *Fragaria virginiana*, native to North America, and *F. chiloensis*, native to South America (Liston et al. 2014; Duchesne 1766; Darrow 1966). Although both progenitor species are indigenous to the Americas, the hybridization event took place in Europe, where subsequent breeding programs during the nineteenth and twentieth centuries facilitated the global expansion of strawberry cultivation (Hernández-Martínez et al. 2023; Darrow 1966; Finn et al. 2013).

Strawberry is valued worldwide for its flavor, nutritional richness, and health-promoting properties, with a global market value projected to surpass USD 43 billion by 2028 (Giampieri et al. 2012; FAO 2023; Priyadarshi et al. 2024). However, the accelerating pace of climate change has emerged as the most formidable threat to the stability of this rapidly expanding industry. Extreme weather fluctuations and persistent global warming are reshaping agroecosystems by weakening plant immune defenses and promoting the emergence and establishment of novel pests and pathogens. As global temperatures continue to rise, the distribution ranges of agricultural pests and plant pathogens are expanding toward higher latitudes and elevations, fundamentally altering long-standing disease dynamics (Schneider et al. 2022). In Europe alone, a total of 142 novel pathogen introductions were recorded between 2012 and 2022, including viruses (37%), bacteria (32%), and fungi (25%) (Raza and Bebber 2022). Elevated temperatures are also associated with increased species richness in several genera of plant pathogens, including *Alternaria*, *Fusarium*, and *Venturia* (Raza and Bebber 2022). Furthermore, climate change is expected to accelerate the spread and establishment of weeds, insect pests, and plant diseases to previously unaffected regions, adding further challenges to sustainable crop production. In addition, climate change contributes to a progressive increase in the frequency and intensity of plant stress events, particularly during the critical phenological stages of flowering, fruit set, and the pre-harvest period. In climate-sensitive regions such as the Mediterranean Basin, future climate scenarios predict a sharp rise in plant pathogens, which are likely to result in significant yield reductions and increased inter-annual variability in production (Trnka et al. 2012).

Strawberry cultivation often relies on heavy application of chemical agents to cope with microbial pathogens. This may lead to the accumulation of pesticide residues and pose potential risks to human health. Currently, up to 36 pesticide applications are used annually, with approximately 18 kg/ha of plant protectant agents applied (Dara 2016; Song et al. 2020; Wardlaw et al. 2023). As a result, strawberries are consistently ranked near the top of the “Dirty Dozen,” a list of the most pesticide-contaminated fruits and vegetables (Winter and Katz 2011). This underscores the urgent need for alternative strategies that significantly reduce the dependency of strawberry production on chemical inputs and thus improve its long-term sustainability. The adoption of hydroponic soil-less cultivation systems of strawberry production that reduce the introduction of soil-borne diseases and pests offers one promising alternative (Hernández-Martínez et al. 2023; Oğuz et al. 2022). Other challenges remain, however, as rising temperatures due to climate change lead to heat stress, while the continuous recirculation of nutrient solutions in closed systems causes salt accumulation within the beds, resulting in salt stress that must also be addressed (Sakamoto et al. 2016; Jamwal and Sharma 2019).

The strawberry holobiome, defined as the ecological and functional unit comprising the host and its associated microbiota, represents a critical determinant of plant health and adaptive capacity under environmental stress. Microbial consortia inhabiting the rhizosphere, phyllosphere, endosphere, and fruit surface contribute to pathogen suppression, modulation of immune responses, nutrient acquisition, and tolerance to drought, salinity, and thermal stress. These functions highlight the holobiome as a central axis of physiological resilience and ecological integration in strawberry cultivation. Recent advances in metagenomics and microbial ecology have revealed intricate interactions within plant-associated microbiomes. However, a comprehensive framework that integrates the biological compartments and applied relevance of the strawberry holobiome remains insufficiently developed. Unlike seed-propagated crops, strawberry reproduces vegetatively through stolons, which connect mother and daughter plants and enable the partial transfer of associated microorganisms. The plant also exhibits a distinctive crown-based architecture, where leaves and roots originate from a compact stem, and develops a receptacle-derived fruit rather than an ovary-derived pericarp. These unique anatomical and reproductive traits may lead to microbiome compositions distinct from those of other crops. Nevertheless, quantitative and system-level investigations of the strawberry holobiome remain limited.

This review discusses the biological distinctiveness of the strawberry holobiome and summarizes the composition of microbial communities reported to date, categorized into the rhizosphere, phyllosphere, and fruit microbiome. In particular, it integrates studies on microbiome modulation under biotic stress, providing important insights into the social networking mechanisms through which strawberry plants interact with surrounding microbiota in response to environmental stimuli. In addition, beneficial microbes capable of alleviating or suppressing biotic stress are introduced, and future directions are proposed for developing holobiome-based strategies to achieve sustainable strawberry cultivation in the era of climate change.

## The genesis and evolution of the holobiome concept

The term holobiome originated from the concept of the holobiont, which was first proposed by the theoretical biologist Adolf Meyer-Abich in 1943 and later developed by Lynn Margulis, who described it as a plant or animal host integrated with its symbiotic microorganisms, forming a tightly associated biological unit throughout much of its life cycle (Amidon 2008; Margulis and Fester 1991). The holobiome concept achieved further prominence with the development of next-generation sequencing technologies, which enabled quantitative analysis of microbial community structure and function. Initially, holobiome referred to the entire community of organisms that constituted the holobiont (the collective sum of their genomes) and was sometimes used interchangeably with hologenome (Guerrero et al. 2013). In recent years, however, the scope of the holobiome has expanded beyond individual host organisms to encompass the broader, interconnected microbial ecosystems that span soil, plants, animals, and humans, reflecting an extended ecological framework. In this review, the term strawberry holobiome indicates a specific group in the microbial community (microbiota) that has evolved to interact with specific plant organs, such as the leaf, root, and fruit, and grow upon (epiphytes) or within (entophytes) strawberry plants.

Recent studies have shown increasing interest in applying plant holobiome concepts to agriculture (Vandenkoornhuyse et al. 2015; Sayyed and Ilyas 2024). Certain plant species, including strawberry, are highly susceptible to many diseases, emphasizing the importance of holobiome-based approaches to their cultivation. The plant holobiome function as integrated biological units in close association with diverse microbial communities, which play essential roles throughout plant development, including pathogen suppression, immune regulation, and mitigation of environmental stresses (Trivedi et al. 2020). Beneficial microbes, in particular, help block pathogen colonization and activate host defense mechanisms, whereas excessive proliferation of pathogenic microbes can impair host physiological functions and increase disease incidence (Mendes et al. 2011; Compant et al. 2025). The balance between beneficial microbiota and the pathobiome has a significant impact on plant health, disease resistance, and fruit quality (Bass et al. 2019), and maintaining this balance is considered a key strategy for enhancing the resilience and sustainability of strawberry cultivation.

The holobiome concept has also introduced a new perspective on traditional breeding strategies. Rather than focusing solely on selecting plant genetic traits, recent approaches employ holobiont-level breeding strategies that consider host–microbe interaction traits, such as the ability to recruit and maintain beneficial microbes (Wei and Jousset 2017; Marco et al. 2022). Some individual plants activate the so-called "cry for help" mechanism, whereby beneficial microbes are actively recruited in response to pathogen attack. Selection for such traits may enhance disease resistance without the need for external chemical inputs (Sharifi and Ryu 2021)**.**

## The uniqueness of the strawberry holobiome

**Fig. 1:**
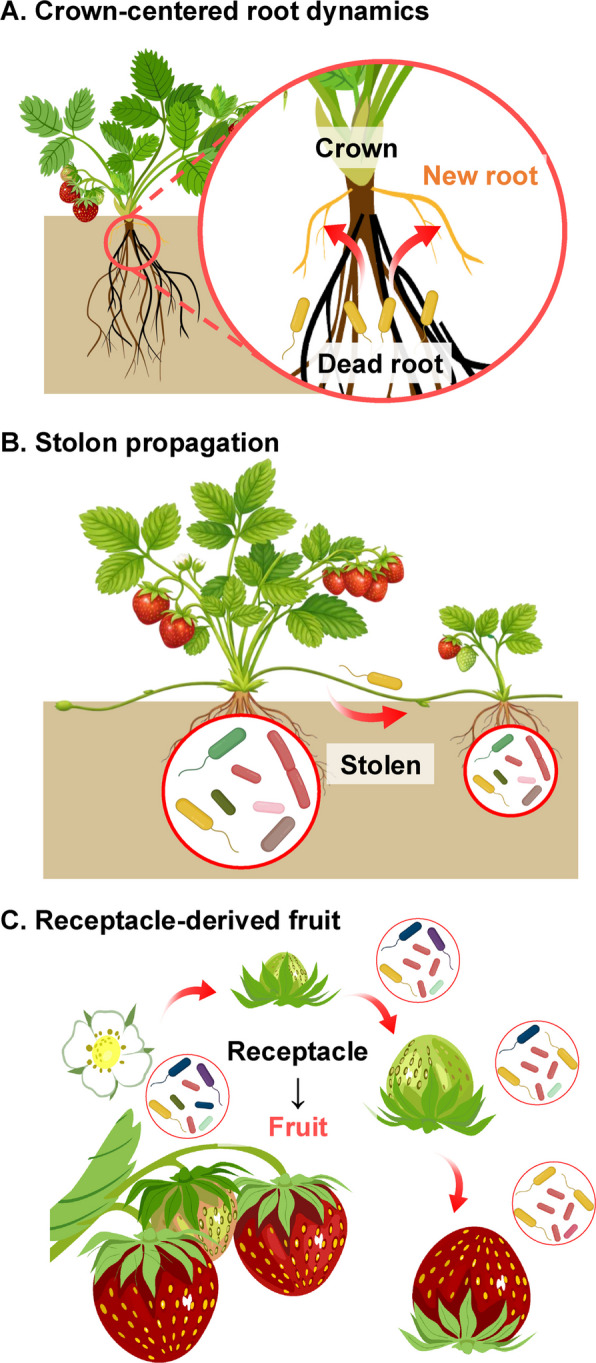
The uniqueness of the strawberry holobiome. **A** Crown-centered root dynamics. Crown-centered root turnover continuously generates new root niches, repeatedly reshaping rhizosphere colonization and microbial succession. **B** Asexual daughter plant formation via stolons provides a physical route for partial holobiome transmission, potentially influencing microbiome assembly in daughter plants. **C** Receptacle-derived fruit. Fruit development from the floral receptacle suggests continuity between flower- and fruit-associated niches, supporting potential microbial transfer and redistribution during fruit formation

Most dicotyledonous plants develop a well-defined taproot system, whereas strawberry possesses a crown-based root architecture in which short-lived adventitious and lateral roots are predominant (Labadie et al. 2023). The crown functions as a central axis connecting the aboveground and belowground organs, where new roots are continuously formed and senesced (Tuomainen et al. 2024) (Fig. 1A). This shallow and fibrous root structure, together with rapid root turnover, generates a dynamic rhizosphere environment characterized by recurrent microbial colonization and succession. These structural characteristics indicate the potential for the formation of a distinctive holobiome, suggesting that the microbial composition and function in the strawberry rhizosphere may exhibit unique ecological patterns (Zhang et al. 2023a).

Strawberry reproduces not only sexually through seeds but also asexually through stolons (runners), suggesting that the holobiome of the mother plant may be partially transmitted to its daughter plants (Fig. 1B). Kukkurainen et al. (2005) analyzed various tissues of *Fragaria* × *ananassa* and *F. vesca* and first reported the presence of identical endophytic bacteria, *Pseudomonas fluorescens* and *Pantoea* spp., in both stolons and seeds. This finding indicated that microorganisms could be vertically transmitted through both clonal propagation via stolons and sexual propagation via seeds. Subsequently, microscopic and PCR analyses confirmed that *Azospirillum brasilense* can migrate from the mother plant to daughter plants through stolons, and this bacterium is presumed to promote the growth of daughter plants through nitrogen fixation and IAA biosynthesis (Guerrero-Molina et al. 2012). However, microbial transmission through stolons is not limited to beneficial bacteria. *Fusarium oxysporum* f. sp. *fragariae* is transmitted via stolons to daughter plants, causing Fusarium wilt, and *Phlomobacter*, the causal pathogen of strawberry marginal chlorosis disease, has also been shown to move through stolons (Pastrana et al. 2019; Dittmer et al. 2021). These findings provide important evidence that plants can transmit microorganisms through stolon-mediated pathways. Although quantitative analyses at the microbiome level remain limited, elucidating the composition and transmission mechanisms of stolon-associated microbial communities will provide key insights into the intergenerational continuity of the holobiome. Such continuity is expected to facilitate the establishment of stable microbial assemblages during the development of daughter plants and to maintain disease resistance and physiological resilience under stress conditions.

Strawberry plants reproduce asexually through stolons rather than seeds, suggesting the holobiome of the mother plant may be partially transmitted to its daughters during vegetative propagation. Although experimental evidence is currently limited, characterizing the microbial communities associated with runners and their potential transfer may provide insight into holobiome continuity across generations. This continuity may not only support the development of healthy daughter plants but also facilitate the establishment of a stable holobiome under stress conditions, potentially enhancing disease resistance.

In addition to its distinctive root architecture and vegetative propagation traits, strawberry displays a unique fruit morphology in which the receptacle, rather than the ovary, enlarges to form the fleshy edible portion (Fait et al. 2008) (Fig. 1C). The true fruits are the achenes distributed across the receptacle surface, and the red flesh that is consumed corresponds to the expanded receptacle tissue rather than the ovary. This anatomical feature produces a fruit surface fundamentally different from the pericarp-derived tissues of typical fruits and provides a nutrient-rich and highly exposed niche favorable for microbial colonization. Therefore, the receptacle-based fruit structure suggests the potential for the formation of a distinctive fruit microbiome specific to strawberry.

Hydroponic cultivation systems are increasingly being adopted to ensure stable production and yield of high-quality strawberries, while traditional soil-based cultivation is gradually declining. This shift in cultivation practices may have a significant impact on the composition and function of the strawberry holobiome. In soil-based systems, the rhizosphere acts as a reservoir for diverse microbial communities; however, in hydroponic systems, microbial input from external sources is limited, potentially leading to the formation of a holobiome that differs from that in soil. Accordingly, elucidating the composition and functional roles of the holobiome under hydroponic conditions, and developing strategies to introduce and stably maintain beneficial microbes in this environment, has emerged as a critical research priority.

The distinctive morphological and physiological characteristics of strawberry suggest the potential formation of a holobiome that is clearly differentiated from that of other crops. These structural and reproductive traits may drive the assembly of specialized microbial communities across multiple plant compartments, including the rhizosphere, phyllosphere, and carposphere. However, the ecological roles and functional significance of the strawberry holobiome remain insufficiently elucidated. It is still unclear whether the holobiome acts as a driving factor directly regulating plant development, growth, and fitness, or whether it simply represents a responsive outcome shaped by plant physiological status and environmental conditions. Future studies integrating multi-omics analysis with experimental validation are required to clarify how the strawberry holobiome contributes to plant growth, health, and environmental adaptability.

## Ecological aspects of the strawberry holobiome

### Rhizosphere holobiome

**Fig. 2:**
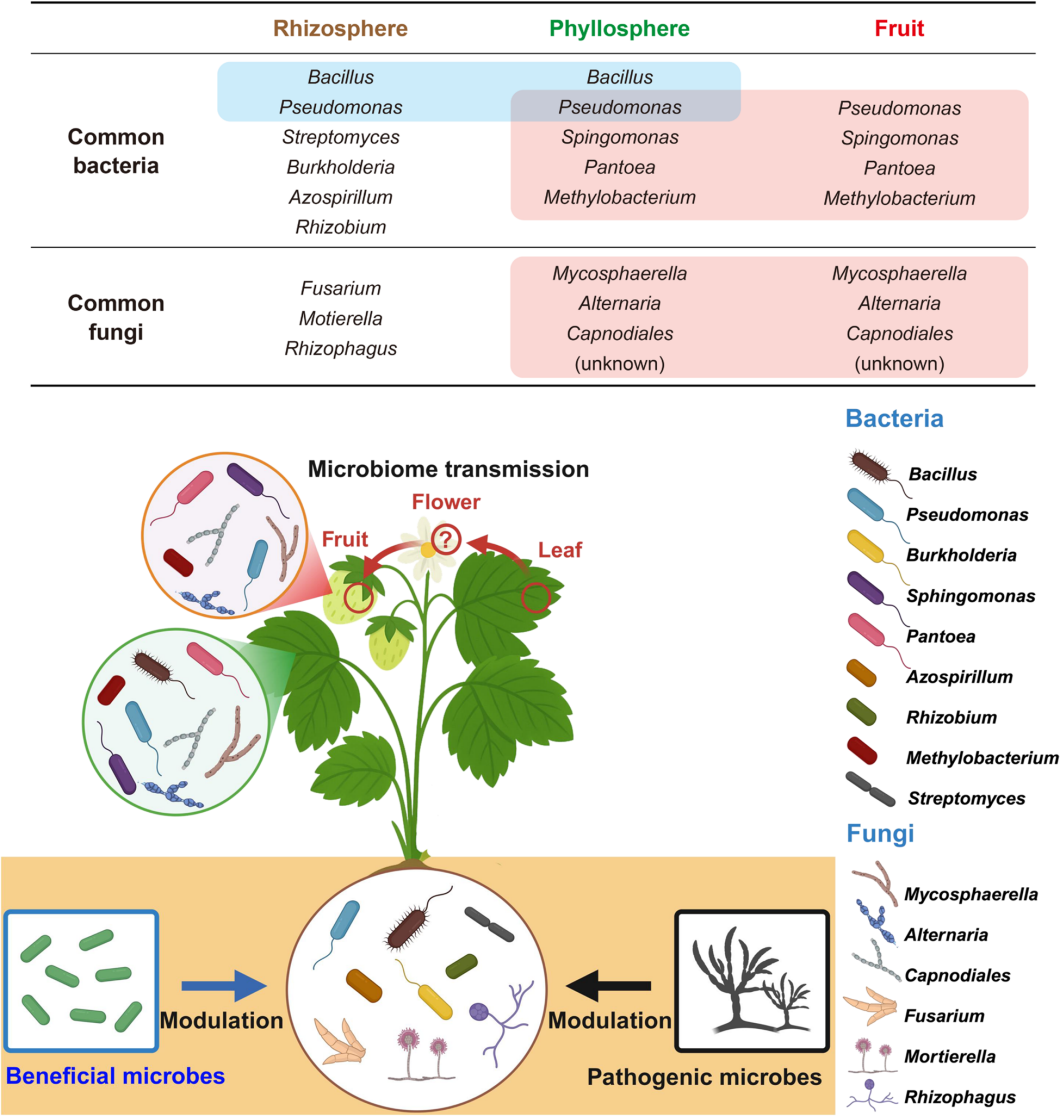
Assembly and modulation of the strawberry-associated microbiome across different plant compartments. This figure illustrates the compartment-specific assembly and modulation of microbial communities across the strawberry holobiome, which is comprised of the rhizosphere, phyllosphere, and fruit. Upper panel: Table summarizing the common microbial taxa detected across compartments. Blue shading indicates microbes shared between the rhizosphere and phyllosphere, while red shading indicates microbes shared between the phyllosphere and fruit. Lower panel: Leaf-associated microbes are presumed to be transferred to developing fruits via floral tissues. Beneficial microbes and pathogens modulate the strawberry microbiome, either supporting plant growth or inducing dysbiosis

The rhizosphere is a front line in the battlefield between pathogens and the beneficial holobiome (microbiome) (Fig. 2). Recent microbiome analyses revealed that the strawberry rhizosphere is dominated by Proteobacteria (45–55%), followed by Actinobacteria (10–30%), Bacteroidetes (5–12%), Acidobacteria (5–10%), and Firmicutes (2–5%) (Olimi et al. 2022; Lazcano et al. 2021; Sangiorgio et al. 2022a). Among these bacterial taxa, the genera *Pseudomonas*, *Bacillus*, *Streptomyces*, *Azospirillum*, and *Rhizobium* are consistently detected as dominant members of the strawberry rhizosphere. The fungal community is mainly composed of Ascomycota (70–90%), followed by Mortierellomycota (5–12%) and Basidiomycota (2–8%) (Sangiorgio et al. 2022b; Hu et al. 2025; Li and Liu 2019). Within Ascomycota, Sordariomycetes (40–60%) is the dominant class, represented by saprophytic genera such as *Fusarium*, *Thelonectria*, and *Chaetomium*. In addition, arbuscular mycorrhizal fungi, including *Rhizophagus* (Glomeromycetes, 1–3%), are also detected in the rhizosphere.

In general, the rhizospheric microbiome of plants is shaped by both biotic and abiotic factors in a complex and dynamic manner. Among biotic factors, pathogen infection and inoculation with beneficial microbes are considered major drivers of microbial community restructuring. In particular, microbial assemblages formed under pathogen-challenging conditions are known to play critical roles in the activation and modulation of host defense mechanisms. A representative example involves the activation of plant immune responses upon pathogen recognition. Pathogen perception signals induce the expression of defense-related transcription factors, such as *MYB72*, which subsequently activate the coumarin biosynthesis pathway. As a result, coumarin derivatives such as sideretin and fraxetin are secreted into the rhizosphere as root exudates (Voges et al. 2019). In parallel, salicylic acid (SA)-dependent signaling pathways trigger the transcription of the *AtALMT1* gene (*Aluminum-activated malate transporter 1*), leading to the release of organic acids such as L-malic acid from the roots (Rudrappa et al. 2008). These phenolic compounds and organic acids function as selective cues that enhance the chemotaxis, colonization, and biofilm formation of beneficial microbes such as *Pseudomonas* and *Bacillus*, thereby contributing to the restructuring of the root microbiome in favor of plant protection (Rudrappa et al. 2008). Nevertheless, the classical view of host–pathogen interactions has largely overlooked the functional contribution of these microbial communities, despite their central role in mediating plant immunity.

#### Modulation of the rhizosphere holobiome by microbial pathogen infection

In 2015, Cha and colleagues conducted a pioneering study elucidating the disease-suppressive function of the rhizosphere microbiome, demonstrating that the microbial community in the strawberry rhizosphere contributes to soil suppressiveness against *Fusarium* wilt (Cha et al. 2015). They identified a field in South Korea that maintained remarkably low disease incidence despite 15 years of continuous strawberry monoculture. Analysis of microbial diversity revealed a strong correlation between the relative abundance of Actinobacteria and disease suppression. Through culture-based isolation, the authors identified *Streptomyces* sp. S4-7, which produces a ribosomally synthesized thiopeptide antibiotic, conprimycin. This compound inhibits *Fusarium oxysporum* by targeting the cell wall remodeling kinase PKC1 and the Golgi-associated transporter SBE2, thereby disrupting cell wall biosynthesis and suppressing pathogen growth.

Following this discovery, subsequent microbiome studies expanded the scope to other soil-borne fungal pathogens affecting strawberry, including *Macrophomina phaseolina* and *Verticillium dahliae* (Lazcano et al. 2021; Hassani et al. 2023) (Fig. 2). Inoculation of *Macrophomina phaseolina* decreased microbial diversity, as indicated by the Shannon index, whereas *Verticillium dahliae* inoculation increased microbial diversity (Lazcano et al. 2021). Although cultivar-dependent recruitment of specific bacterial taxa was observed, cultivars resistant to *Verticillium dahliae* showed a consistent enrichment of Actinobacteria, including *Arthrobacter*, *Nocardioides*, and *Gaiella*, as well as Acidobacteria. These taxa are presumed to contribute to the suppression of *Verticillium dahliae* through antifungal activity and the production of cell wall–degrading enzymes such as chitinase. In contrast, during *Macrophomina phaseolina* infection, Actinobacteria, including *Streptomyces* and *Nonomuraea*, together with *Pseudomonas* (Proteobacteria), were enriched and are likely to inhibit pathogen growth by producing antifungal metabolites and siderophores (Lazcano et al. 2021). Subsequently, the same research group conducted a field experiment using a *Macrophomina phaseolina*–strawberry interaction model with three cultivars (two resistant and one susceptible) (Hassani et al. 2023). They reconfirmed that Actinobacteria, including *Streptomyces*, and Proteobacteria, including *Pseudomonas*, were dominant in the resistant cultivars, consistent with previous findings. In addition, beneficial microbial taxa such as *Mesorhizobium*, *Sphingomonas*, *Novosphingomonas*, *Nocardioides*, and *Mucilaginibacter*, which interact with plants and promote growth, were enriched in the resistant cultivars.

*Verticillium dahliae* inoculation significantly reduced fungal OTU richness in both the rhizosphere and root compartments. In the rhizosphere, pathogen-associated or saprotrophic fungi such as *Cryptococcus*, *Phoma*, and *Mortierella* increased, whereas the root compartment exhibited a pronounced enrichment of *Leptodontidium* sp. C2 BESC319g, a fungus with potential antifungal activity. This shift was more evident in the resistant cultivar Florence than in the susceptible cultivar Honeoye, with antifungal taxa such as *Leptodontidium* and *Trichosporon* being selectively enriched (Nallanchakravarthula et al. 2014).

In addition to soil-borne pathogens, air-borne microbial pathogens also influence rhizosphere microbiome modulation. Foliar challenge of strawberry leaves by the gray mold *Botrytis cinerea* altered the root microbiome by enriching Actinobacteria while reducing Acidobacteria (De Tender et al. 2016). In contrast, *Colletotrichum* infection increased both bacterial and fungal diversity and richness, accompanied by a decline in beneficial microbes such as *Streptomyces*, *Bacillus*, *Azospirillum*, and *Trichoderma*, and an enrichment of potential opportunistic or pathogenic taxa including *Pseudomonas*, *Rhodococcus*, and *Fusarium* (Su et al. 2022). These findings suggest that pathogen infections can reshape the rhizosphere microbiome, potentially influencing belowground ecological interactions. However, further research is needed to determine whether these changes in the rhizosphere microbiome represent a plant-driven response that recruits beneficial microbes for defense or a pathogen-mediated interference that disrupts plant immune signaling.

In summary, microbial pathogen infection induces modulation of the strawberry rhizosphere holobiome (Fig. 2). However, the extent and direction of these changes appear to depend on the plant’s genetic background and resistance level. Therefore, further mechanistic studies are required to elucidate how host resistance is linked to the assembly and functional reorganization of the rhizosphere holobiome.

#### Modulation of the rhizosphere holobiome by plant-beneficial microbes

The introduction of plant-beneficial microbes provides a promising approach for improving crop performance by modulating the microbial communities in the rhizosphere (Fig. 2). Such microbial inputs operate not only through direct colonization but also indirectly by restructuring the native microbial networks via ecological interactions with the host plant. Nam and colleagues (Nam et al. 2023) applied a consortium of plant growth-promoting rhizobacteria (PGPR) composed of *Bacillus subtilis*, *Bacillus amyloliquefaciens*, and *Pseudomonas monteilii* to field-grown strawberry plants. The PGPR treatment selectively enriched key functional taxa in the rhizosphere, resulting in a functional reorganization of the microbial network. The relative abundances of *Bacillus* and *Pseudomonas* increased, along with beneficial microbes such as *Roseiarcus*, *Rhodanobacter*, *Devosia*, *Microvirga*, *Ramlibacter*, *Flavobacterium*, and *Variovorax*, which are involved in nitrogen fixation, nutrient cycling, pathogen suppression, and plant growth promotion. Although α- and β-diversity remained largely unchanged, the relative composition of functionally important microbial groups shifted, indicating a restructuring of the rhizosphere microbiome toward greater functional stability.

VESTA (SOBEC Corporation, Fowler, CA) is a commercially available fermented microbial soil amendment composed of a complex mixture of diverse bacteria, fermentation by-products, and organic acids, including *Mycobacterium*, *Caulobacter*, *Novosphingobium*, *Bacillus*, *Flavobacterium*, and *Pseudomonas*. Application of VESTA to strawberry plants grown in open-field conditions decreased Shannon diversity (α-diversity) in soil and rhizosphere, while increasing β-diversity, indicating greater compositional heterogeneity between the treated and control microbiomes (Deng et al. 2019). Taxonomic analysis revealed a significant enrichment of Betaproteobacteria, particularly *Ramlibacter*, *Comamonadaceae*, *Acidovorax*, and *Methylophilaceae*, which are known to participate in nitrogen fixation, denitrification, and sulfur cycling. Interestingly, most of the taxa that increased in abundance after treatment were not included in the VESTA formulation. This indicates that VESTA did not directly replace the native microbial community but instead induced a rewiring of pre-existing microbial networks mediated by plant–microbe interactions.

#### Modulation of the rhizosphere holobiome by hydroponic cultivation

The increasing use of hydroponic systems for strawberry cultivation means that understanding how artificial substrates reshape the root microbiome from that found in conventional soil-based systems has become essential. A recent study characterized the structural and functional dynamics of the root microbiome in a strawberry cultivar, Benihope, cultivated in greenhouse soil and two different artificial substrates (Zhang et al. 2023b). β-diversity analysis revealed a clear separation in root bacterial community composition between the artificial substrates and soil; significant differences were also observed between the two substrates, indicating substrate-specific microbial selection; moreover, α-diversity indices were significantly higher in both artificial substrates than in soil. At the compositional level, genera such as *Burkholderia*, *Acidocella*, *Asticcacaulis*, and *Turicibacter* were enriched in plants grown in artificial substrates, whereas *Streptomyces* and *Cellvibrio* were significantly reduced.

These shifts were accompanied by functional changes in the root microbiome. In silico functional prediction indicated significant upregulation of metabolic pathways associated with flavonoid and flavanol biosynthesis in artificial substrates, while pathways related to the biosynthesis of antimicrobial compounds, including clavulanic acid, stilbenoid, and macrolides, were downregulated. These findings suggest that hydroponic conditions may reduce the microbe-mediated suppressive potential against invading pathogens, thereby increasing disease susceptibility in strawberry plants grown in artificial substrate-based systems.

### Phyllosphere holobiome

The phyllosphere, encompassing aerial plant organs such as leaves, stems, flowers, and fruits, provides a habitat for diverse microbial communities including bacteria, fungi, viruses, and protozoa (Sohrabi et al. 2023). Particularly, the phyllosphere serves as an ecological interface that directly interacts with various arthropods, including pollinators and predatory mites, functioning as an important conduit for microbial introduction and dissemination (Kim et al. 2019; Legein et al. 2022). Among these tissues, leaves are the most intensively studied because of their direct exposure to environmental stress and distinctive surface structure. Phyllosphere microbes have evolved adaptations such as the production of pigments, extracellular polysaccharides, and biosurfactants to cope with ultraviolet radiation, desiccation, and nutrient limitation. They can also modulate or evade host immune responses, enabling stable colonization of above-ground tissues (Bashir et al. 2022; Sohrabi et al. 2023; Thapa and Prasanna 2018).

Recent studies have revealed that phyllosphere microbes are not passive epiphytes but active biological agents that influence plant physiology, growth, immunity, and stress tolerance (Bashir et al. 2022; Sohrabi et al. 2023; Stone et al. 2018). Their roles are comparable to those of PGPR in the rhizosphere. However, the phyllosphere holobiome of strawberry plants remains less characterized than the root- and soil-associated microbiomes. Most studies have focused on taxonomic variation among cultivars, while the functional relevance of these microbial assemblages to host performance is still poorly understood.

Across strawberry cultivars, bacterial diversity in the phyllosphere is generally lower than in root-associated compartments, whereas fungal diversity remains relatively stable among tissues. In the cultivars Elsanta and Darselect, Actinobacteria dominate the phyllosphere, while cultivar Monterey is enriched in Gammaproteobacteria. Fungal communities are comparatively conserved, with Dothideomycetes being the most abundant class, followed by Tremellomycetes, Leotiomycetes, and Sordariomycetes. Functional predictions suggest that *Methylobacterium*, known for its plant-beneficial properties, is highly enriched in the above-ground tissues of Monterey and Darselect (Sangiorgio et al. 2022a).

Other cultivars including Mara des Bois, White Ananas, White Elves, Tokun, and Akihime also exhibit clear genotype- and tissue-dependent differentiation (Olimi et al. 2022) (Yang et al. 2025). A consistent core bacterial community dominated by *Sphingomonas*, *Pseudomonas*, *Bacillus*, *Methylobacterium*, and *Flavobacterium* has been identified across these cultivars, while dominant fungal genera include *Mycosphaerella*, *Alternaria*, and *Helotiales*. In White Elves, microbial communities were functionally associated with cell growth, motility, and energy metabolism, whereas in Akihime, predicted functions were related to amino acid metabolism and xenobiotic degradation, suggesting genotype-specific microbial adaptation. These functions were inferred from in silico analyses and require validation through metatranscriptomic, proteomic, or metabolomic studies to confirm their ecological significance.

The phyllosphere, including flowers and leaves, directly interacts with various arthropods such as pollinators and predatory mites, resulting in potential modulation of the phyllosphere microbiome. Notably, the detection of *Buchnera* populations, typically associated with aphids, in the aerial phyllosphere including fruits suggests that above-ground tissues can serve as an ecological interface that closely interacts with insects (Olimi et al. 2022). This concept is supported by the findings of Legein et al. (2022), who reported that the introduction of predatory mites led to an enrichment of *Sphingomonas*, *Methylobacterium*, and *Pseudomonas* in the strawberry phyllosphere. Strawberry plants also maintain intimate associations with pollinators. Pollinating bees can introduce *Snodgrasella* and *Gilliamella* to the phyllosphere (Legein et al. 2022). In addition to introducing external microbes, bee activity also mediates the transmission of beneficial bacteria already residing in the phyllosphere. *Streptomyces globisporus* SP6C4, originally detected in the strawberry phyllosphere, has been shown to spread among plants through pollinator movement (Kim et al. 2019). This bacterium suppresses fungal diseases by inhibiting phytopathogenic fungi and also blocks the invasion of entomopathogenic fungi within bees. Interestingly, SP6C4 exhibits 99% genome similarity to the rhizosphere-derived beneficial strain *S. globisporus* S4-7, implying that beneficial rhizobacteria can migrate to aerial tissues and participate in a bacteria–plant–insect tripartite interaction that contributes to disease suppression and ecosystem stability.

The leaf microbiome can be transferred to strawberry fruit (Olimi et al. 2022). The microbial communities of leaves and fruits share similar taxa, indicating continuous microbial transfer during fruit development. Fungal transmission rates (50–99%) are considerably higher than those of bacteria (38–80%), whereas microbial movement from the root and rhizosphere to the fruit is very limited. This highlights the phyllosphere as a key microbial reservoir influencing fruit health, disease resistance, postharvest quality, and microbiome-based management strategies in strawberry cultivation.

### Fruit holobiome

Compared with the phyllosphere, studies of the strawberry fruit microbiome during the pre-harvest stage are scarce. Most research has focused on post-harvest conditions, particularly on microbiomic shifts following pathogen infection or application of biological control agents. To date, the study by Olimi et al*.* (2022) remains the only detailed investigation of the fruit microbiome during fruit development. Their results showed a clear separation between below-ground (root and rhizosphere) and above-ground (leaf and flower) microbial communities. A high proportion of bacteria was transmitted among above-ground organs, whereas only a small fraction originated from the rhizosphere (Fig. 2). In contrast to the strong cultivar-dependent variation observed in the rhizosphere and phyllosphere, the fruit microbiome exhibited limited cultivar-specific differences. The dominant bacterial genera included *Sphingomonas*, *Pseudomonas*, *Pantoea*, *Methylobacterium*, and *Massilia*, whereas *Mycosphaerella*, *Alternaria*, and unidentified *Capnodiales* were the major fungal taxa. As the fruit ripened, bacterial abundance and diversity increased, while fungal changes were relatively modest. During storage, *Methylobacterium* abundance increased, while *Sphingomonas* declined. Among fungi, *Mycosphaerella* and *Alternaria* increased, but *Capnodiales* decreased. After *Botrytis cinerea* infection, both bacterial and fungal abundances increased, but overall microbial diversity declined sharply, with *Botrytis* becoming dominant. These findings, however, were derived from a single study, underscoring the need for additional research to clarify the ecological and functional dynamics of fruit-associated microbiomes.

Recent studies have highlighted the role of microbial biocontrol agents in modulating the strawberry fruit microbiome during post-harvest storage. Application of *Debaryomyces hansenii* increased fungal α-diversity while reducing the relative abundance of *Cladosporium*, although *Botrytis* abundance rose during later storage (Zhao et al. 2023). Similarly, pre-harvest treatment with *Metschnikowia fructicola* enriched beneficial bacterial genera such as *Methylobacterium*, *Sphingomonas*, *Rhizobium*, and *Bacillus*, while suppressing the pathogenic fungus *Botrytis* (Zhimo et al. 2021). These findings suggest that microbial biocontrol agents not only inhibit pathogens directly but also reshape the fruit microbiome, contributing to disease suppression and maintenance of post-harvest fruit quality.

Beyond disease control, the fruit-associated microbiome plays a crucial role in shaping the nutritional and postharvest traits of strawberry fruit. Beneficial microorganisms can influence sugar–acid balance, pigment accumulation, and volatile compound biosynthesis, thereby enhancing sweetness, color, and aroma quality (Todeschini et al. 2018). Collectively, these findings suggest that the strawberry fruit holobiome contributes not only to disease suppression but also to the edible quality and commercial value of the fruit. Future studies should investigate how targeted manipulation of the fruit microbiome can be applied to enhance flavor, extend shelf life, and improve the overall market value of strawberries.

## Microbial protection of strawberry against microbial pathogens

**Fig. 3:**
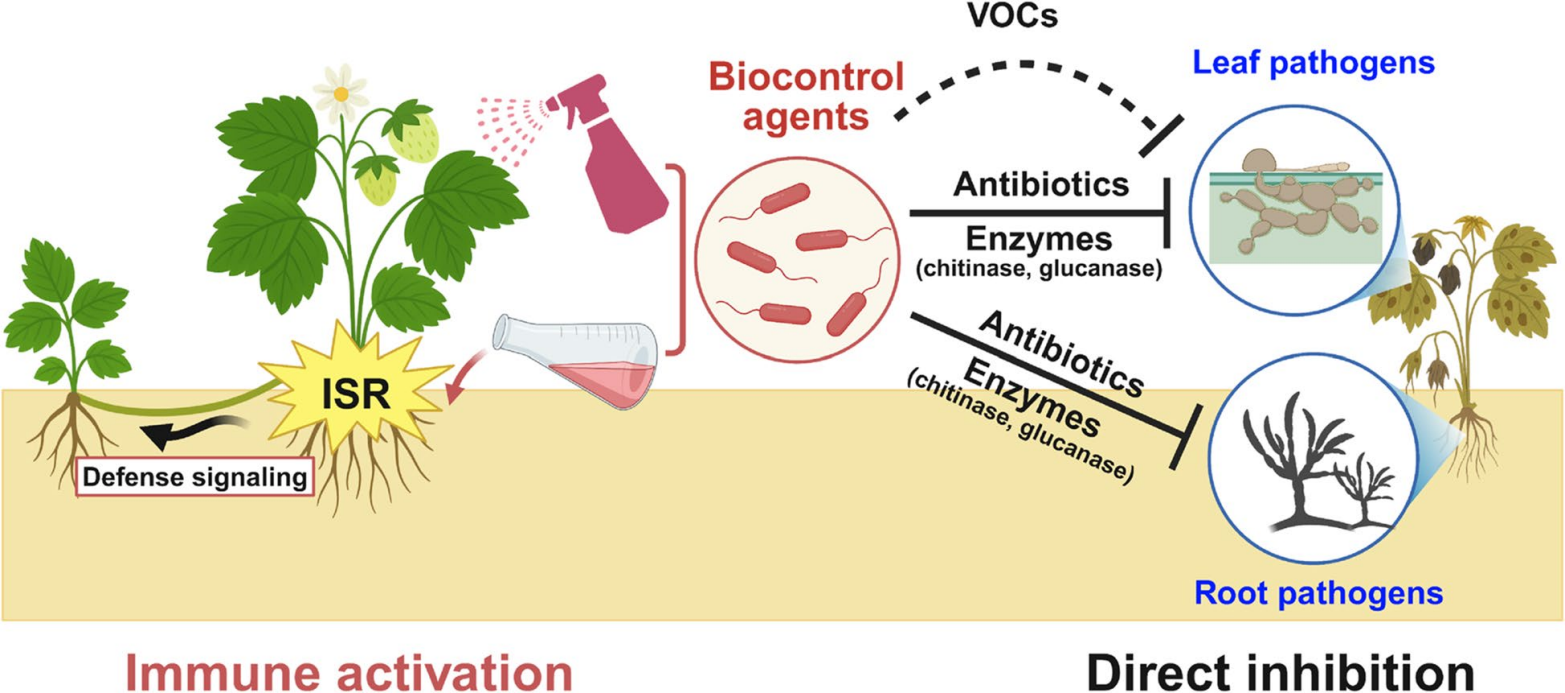
Modes of action of microbial biocontrol agents against strawberry pathogens. Microbial biocontrol agents suppress strawberry pathogens through both direct and indirect mechanisms. Direct inhibition involves the production of antibiotics, cell wall-degrading enzymes such as chitinases and glucanases, and volatile organic compounds (VOCs) that target microbial pathogens associated with both roots and leaves. In addition, a subset of biocontrol agents induces systemic resistance (ISR) in the host plant, thereby priming immune responses against subsequent pathogen challenges. ISR-triggered defense signaling influences not only the treated mother plant but also its runner-derived daughter plants

Practical approaches to disease management are currently limited to applications of a single microbe rather than a microbial community, even though recent studies show strawberry plants provide habitats for diverse microbes. In this section, we discuss disease control methodologies involving environmentally friendly biological and chemical agents against microbial pathogens of the aerial and below-ground organs of strawberry plants (Fig. 3 and Table 1).

**Table 1:** Biological control agents for strawberry leaves and fruit and their modes of action

Infection sites	Diseases (pathogen species)	Biocontrol agents	Mechanisms	References
Leaves	Anthracnose (*Colletotrichum* spp.)	*Bacillus amyloliquefaciens* S13-3	Direct inhibition by antifungal compounds including iturin A	Mochizuki et al. (2012)
		*Bacillus atrophaeus* DM6120	Direct inhibition by antifungal compounds	Alijani et al. (2022)
		*Paenibacillus polymyxa* TP3	Direct inhibition by antifungal compounds, and Induction of systemic resistance via jasmonic acid-dependent signaling	Lee et al. (2024)
		*Bacillus amyloliquefaciens* PMB05	enhancement of antioxidant enzyme activity and callose accumulation	Wu et al. (2021)
	Powdery mildew (*Podosphaera aphanis)*	*Penicillium oxalicum*	Direct inhibition of fungal pathogen	De Cal et al. (2008)
		*Trichoderma harzianum* T39, *Rhodotorula* sp. Y13 and *Pseudomonas* sp. B52	Induction of pathways associated with salicylic acid and jasmonic acid	Harel et al. (2011)
		*Ampelomyces quisqualis*, *Trichoderma harzianum* T39 and *Bacillus subtilis* QST713	Direct inhibition of fungal growth and induced systemic resistance	Pertot et al. (2008)
	Neopestalotiopsis leaf spot (*Neopestalotiopsis clavispora*)	*Trichoderma asperellum*	Direct inhibition of fungal growth	Amrutha and Vijayaraghavan (2018)
		*Bacillus cereus* Bce-2	Direct inhibition of fungal growth and induction of defense-related gene expression in strawberry	Zhang et al. (2024)
Fruits	Anthracnose (*Colletotrichum spp.*)	*Bacillus amyloliquefaciens* PMB05	Direct inhibition of fungal growth	Wu et al. (2021)
		*Bacillus velezensis* 12Y	Direct inhibition of fungal growth	Yang et al. (2024a, b)
		*Streptomyces sp.* H4	Direct inhibition of fungal growth	Li et al. (2021a, b)
		*Streptomyces corchorusii* CG-G2	Direct inhibition of fungal growth	Li et al. (2024)
		*Burkholderia sola* NAU20	Direct inhibition of fungal growth	Wang et al. (2025)
	Gray mold (*Botrytis cinerea*)	*Galactomyces candidum* JYC1146	Direct inhibition of fungal growth	Chen et al. (2018)
		*Rhodotorula glutinis*	Direct inhibition of fungal growth	Zhang et al. (2007)
		*Clonostachys rosea*	Direct inhibition of fungal growth	Cota et al. (2008)
		*Bacillus halotolerans* KLBC XJ-5	Direct inhibition of fungal growth and induction of plant resistance	Wang et al. (2021)
	*Other fruit disease*	*Debaryomyces hansenii*	Inhibition of fungal spore germination and activation of antioxidant enzymes and resistance-related metabolites in strawberry	Zhao et al. (2022) Zhao et al. (2023)

### Biological control against soil-borne pathogens

Fusarium wilt, caused by *Fusarium oxysporum* f. sp. *fragariae*, is one of the most destructive soil-borne diseases affecting cultivated strawberry plants, requiring effective and sustainable control strategies. Several *Bacillus* strains have shown potent antifungal activity against this pathogen. *Bacillus velezensis* BS87 and RK1 reduced disease incidence by up to 69.2% under both pot and field conditions (Nam et al. 2009), while *Bacillus subtilis* TS06 inhibited *Fusarium oxysporum* and *Verticillium dahliae* by > 80% in vitro and achieved comparable suppression in pot experiments (Zhang et al. 2012). A bioorganic fertilizer containing *Bacillus licheniformis* X-1 and *Bacillus methylotrophicus* Z-1 suppressed Fusarium wilt by 80%, while enhancing the activities of superoxide dismutase (SOD), peroxidase (POD), polyphenol oxidase (PPO), and catalase (CAT), thereby reducing tissue damage and inducing systemic resistance (Chen et al. 2020).

Beyond bacterial agents, alternative biological approaches have demonstrated durable effects. The microalga *Chlorella fusca* CHK0059 reduced disease severity by 70.4% and pathogen density by 86.8% at 69 days after treatment, confirming its long-term stability (Kim et al. 2020). Co-application of the plant biostimulant 5-aminolevulinic acid (ALA) and *Chlorella* enhanced inhibition of *Fusarium oxysporum* by 43.1% compared with individual treatments, while *Trichoderma harzianum* proliferation was simultaneously promoted, suggesting synergistic activity (Yang et al. 2024a). The arbuscular mycorrhizal fungus (AMF) *Glomus mosseae* increased lignin and hydroxyproline-rich glycoprotein accumulation, enhanced antioxidant enzyme activities, and stabilized cell membranes, conferring systemic resistance to Fusarium wilt (Yanan et al. 2015).

Other soil-borne pathogens, including *Verticillium dahliae*, *Phytophthora cactorum*, and *Macrophomina phaseolina*, also pose serious threats to strawberry production.

Application of *Serratia plymuthica* HRO-C48 reduced *Verticillium* wilt incidence by 24.2% in multi-year field trials and increased fruit yield by 296% (Kurze et al. 2001). Commercial AMF products containing *Glomus* spp. (Agrauxine formulation) significantly suppressed *Verticillium dahliae* and improved plant water status by increasing stomatal conductance, transpiration rate, and leaf water potential, particularly in susceptible cultivars (Sowik et al. 2016).

Several microbial agents demonstrate significant efficacy against *Phytophthora* root and crown rots. *Serratia plymuthica* HRO-C48 reduced *Phytophthora cactorum* incidence by 9.6% in field trials (Kurze et al. 2001). *Pseudomonas fluorescens* EPS817 and EPS894 produce a suite of antimicrobial compounds, including 2,4-diacetylphloroglucinol and phenazine-1-carboxylic acid, that suppressed spore germination by 60% and reduced disease incidence by up to 80% when the two strains are co-applied (Agusti et al. 2011). Similarly, *Raoultella terrigena* G-584, *Bacillus amyloliquefaciens* G-V1, and *Pseudomonas fluorescens* 2R1-7 inhibited *Phytophthora* spp. in vitro and reduced disease severity by 51.5% under greenhouse and field conditions (Anandhakumar and Zeller 2004). Long-term applications of *Trichoderma harzianum* and *Trichoderma viride*, especially when combined with soil solarization, decreased *P. cactorum* density by 99% and disease incidence by 76.6%, maintaining effective rhizosphere colonization over successive seasons (Porras et al. 2007).

*Macrophomina phaseolina*, the causal agent of charcoal rot, has also been successfully controlled using microbial antagonists. Preventive application of *Trichoderma asperellum* T18 reduced disease incidence by 65% in the field and inhibited mycelial growth by 36% in vitro (Pastrana et al. 2016). The biocontrol formulation Fusbact®, composed of *Bacillus megaterium* and *Bacillus laterosporus*, exhibited similar suppression, although performance varied with environmental conditions (Pastrana et al. 2016). Generalist *Trichoderma* strains (*T. harzianum*, *T. album*, *T. viride*) showed broad-spectrum activity against *Rhizoctonia solani*, *Fusarium* spp., and *Macrophomina phaseolina*, reducing root rot incidence by 77%, increasing yield by 36.8%, and improving chlorophyll, phenol, sugar, and protein content (Ahmed and El-Fiki 2017). *Azospirillum brasilense* strains REC3 and 2A1 reduced disease severity by 15–22.5% via induced resistance mechanisms, including stomatal closure and deposition of callose and lignin (Viejobueno et al. 2021a). *Brevibacterium* sp. Hvs8 suppressed charcoal rot by 80%, decreased crown symptom severity by 85%, and reduced plant mortality by 65% under field conditions through induced systemic resistance (ISR)-related structural and biochemical defense activation (Viejobueno et al. 2021b).

Collectively, these findings demonstrate that diverse microbial agents including *Bacillus*, *Trichoderma*, *Serratia*, *Pseudomonas*, AMF *Glomus* spp., *Chlorella*, and beneficial bacteria such as *Azospirillum* and *Brevibacterium* provide broad and sustainable protection against soil-borne strawberry pathogens (Table 2). Their effectiveness derives from both direct antagonism through the production of antifungal metabolites, competitive exclusion, and volatile organic compounds, and indirect plant-mediated mechanisms involving the activation of antioxidant enzymes, ISR, and improved physiological resilience.

**Table 2:** Biological control agents for strawberry roots and their modes of action

Infection site	Disease (pathogen species)	Biocontrol agents	Mechanisms	References
Roots	*Fusarium wilt* (*Fusarium oxysporum* f. sp. *fragariae*)	*Streptomyces* sp. S4-7	Direct inhibition of fungal growth by producing thiopeptide antibiotic conprimycin	Cha et al. (2015)
		*Bacillus velezensis* BS87 and *B. velezensis* RK1	Direct inhibition of fungal growth	Nam et al. (2009)
		*Bacillus subtilis* TS06	Direct inhibition of fungal growth	Zhang et al. (2012)
		*Bacillus licheniformis* X-1 and *B. methylotrophicus* Z-1	Enhancement of plant antioxidant enzyme activity and induction of systemic resistance	Chen et al. (2020)
		*Chlorella fusca* CHK0059	Not elucidated	Kim et al. (2020)
		*Trichoderma harzianum*	Not elucidated	Yang et al. (2024a, b)
		*Glomus mosseae*	Enhancement of plant antioxidant enzyme activity and accumulation of lignin and hydroxyproline-rich glycoproteins	Yanan et al. (2015)
	Verticillium wilt (*Verticillium dahliae*)	*Serratia plymuthica* HRO-C48	Direct inhibition of fungal growth	Kurze et al. (2001)
		*Glomus* spp.	Increase of water contents of strawberry plants	Sowik et al. (2016)
	*Phytophthora* root and crown rots (*Phytophthora cactorum*)	*Serratia plymuthica* HRO-C48	Direct inhibition of fungal growth	Kurze et al. (2001)
		*Pseudomonas fluorescens* EPS817 and EPS894	Direct inhibition of fungal growth	Agusti et al. (2011)
		*Raoultella terrigena* G-584, *Bacillus amyloliquefaciens* G-V1 and *P. fluorescens* 2R1-7	Not elucidated	Anandhakumar and Zeller. (2004)
		*Trichoderma harzianum* and *T. viride*	Not elucidated	Porras et al. (2007)
	Charcoal rot (*Macrophomina phaseolina*)	*Trichoderma asperellum* T18	Direct inhibition of fungal growth	Pastrana et al. (2016)
		*Bacillus megaterium* and *B. laterosporus*	Direct inhibition of fungal growth	Pastrana et al. (2016)
		*Trichoderma* spp.	Direct inhibition of fungal growth	Ahmed and El-Fiki. (2017)
		*Azospirillum brasilense* REC3 and 2A1	Induced systemic resistance	Viejobueno et al. (2021b, a)
		*Brevibacterium* sp. Hvs8	Induced systemic resistance	Viejobueno et al. (2021b, a)

### Biological control against air-borne pathogens

Biological control of foliar diseases in strawberry plants has primarily focused on antagonistic bacteria, particularly those in the genus *Bacillus*, although several fungal and yeast-based antagonists have also been investigated. These agents suppressed pathogen development either by producing antifungal compounds or by inducing host resistance mechanisms such as ISR.

Among the air-borne fungal pathogens affecting strawberry foliage, *Colletotrichum* species, the causal agents of anthracnose, are the most extensively studied targets for biological control. Multiple *Bacillus* strains have demonstrated potent antagonistic activity, enabling the management of anthracnose caused by *Colletotrichum* species. *Bacillus amyloliquefaciens* S13-3, which produces antifungal compounds including iturin A, reduced disease severity by 41.8% in detached leaf assays (Mochizuki et al. 2012). *Bacillus atrophaeus* DM6120, an endophyte isolated from strawberry roots, inhibited mycelial growth and spore germination of *Colletotrichum nymphaeae* by 54.92% and 80.80%, respectively, and provided 94.44% and 88.88% disease suppression when applied via soil drenching or foliar spraying (Alijani et al. 2022). In addition to its direct antifungal effects, *Paenibacillus polymyxa* TP3 suppressed *Colletotrichum siamense* through fusaricidin production. *P. polymyxa* TP3 also upregulated the jasmonic acid-responsive gene *FaPDF1.2* to activate ISR, inducing callose deposition and, ultimately reducing lesion size by 56.8%. Notably, the defense signaling pathway induced by TP3 in mother plants was transmitted to runner-derived daughter plants, resulting in comparable reductions in anthracnose severity, suggesting that IR was maintained across clonal generations (Lee et al. 2024). Similarly, *Bacillus amyloliquefaciens* PMB05 enhanced host antioxidant enzyme activity and induced callose accumulation, reducing disease incidence from 93.3% to 33.3% (Wu et al. 2021).

Microbial antagonists have also been effective against powdery mildew, another major foliar disease of strawberry. *Penicillium oxalicum* reduced disease progress by 25.3–86.9 percent in growth-chamber assays and 25–50 percent in three-year field trials, with efficacy varying among cultivars (De Cal et al. 2008). Additional antagonists such as *Trichoderma harzianum* T39, *Pseudomonas* sp. B52, and *Rhodotorula* sp. Y13 reduced disease severity by 30–60 percent. *Trichoderma harzianum* T39 also induced systemic resistance by up-regulating defense genes including *PR1*, *PR10*, *WRKY*, and *LOX* (Harel et al. 2011). Integrating microbial treatments with chemical fungicides further improves disease control while reducing fungicide residues. Combined treatments involving *Ampelomyces quisqualis*, *Trichoderma harzianum* T39, and *Bacillus subtilis* QST713, especially in rotation with azoxystrobin or penconazole, yielded superior disease suppression compared with biological control alone (Pertot et al. 2008).

The emerging pathogen *Neopestalotiopsis clavispora* has recently been managed using *Trichoderma asperellum*, which reduced disease severity by 75.8 percent (Amrutha and Vijayaraghavan 2018). *Bacillus cereus* Bce-2 inhibited fungal growth by 79 percent in vitro and reduced disease severity by 57.9 percent in field conditions while enhancing the activities of peroxidase, SOD, CAT, and phenylalanine ammonia-lyase (PAL). It also activated multiple defense pathways through up-regulation of *WRKY22*, *WRKY29*, *ChiB*, *RBOHD*, *HSP90*, *RD19*, *PR1*, and *PP2C* (Zhang et al. 2024).

Despite these advances, the biological control of angular leaf spot, caused by *Xanthomonas fragariae* and responsible for yield losses ranging from 8 to 80 percent, remains largely unexplored (Roberts et al. 1997). Considering its economic impact, the development of effective microbial strategies against this disease should be prioritized.

### Biological control of fruit pathogens

The high moisture and carbohydrate contents of strawberry fruit, combined with its delicate texture, make it highly vulnerable to microbial infection during harvest and storage.

Anthracnose, caused by *Colletotrichum* species, is one of the most destructive post-harvest diseases. Among the antagonistic microbes studied, strains of *Bacillus* and *Streptomyces* have shown the most consistent and potent antifungal activity against *Colletotrichum* species. *Bacillus amyloliquefaciens* PMB05 reduced anthracnose incidence from 86.7 to 43.3 percent by inducing spore death through strong antifungal activity (Wu et al. 2021). The marine-derived *Bacillus velezensis* strain 12Y exhibited broad-spectrum antifungal activity and enhanced fruit resistance by activating PAL, cinnamate 4-hydroxylase (C4H), and 4-coumarate:CoA ligase (4CL), leading to increased accumulation of phenolic compounds and anthocyanins without affecting fruit quality (Yang et al. 2024b). Similarly, *Streptomyces* strains have displayed remarkable inhibition of *Colletotrichum* species through both direct and indirect mechanisms. *Streptomyces* sp. H4 inhibited mycelial growth of *Colletotrichum fragariae* by 80.7 percent and completely suppressed disease at higher concentrations by blocking spore germination, inducing hyphal deformation, and disrupting fungal metabolism (Li et al. 2021a). *Streptomyces corchorusii* CG-G2 decreased disease incidence from 90.5 to 9.5 percent and inhibited conidial germination by 99.05 percent via the production of volatile organic compounds (VOCs). These VOCs also stimulated flavonoid biosynthesis and the accumulation of pinocembrin, contributing to enhanced host resistance and improved fruit quality (Li et al. 2024). Other antagonists such as *Burkholderia sola* NAU20 emit VOCs that suppress *Colletotrichum gloeosporioides* by inhibiting mycelial growth and spore germination by 73.2 and 85.7 percent, respectively. Ethyl trans-2-octenoate, one of the VOCs produced, showed the highest antifungal activity and activated the flavonoid and phenylpropanoid biosynthesis pathways in fruit tissues (Wang et al. 2025).

Gray mold, primarily caused by *Botrytis cinerea*, is another major post-harvest disease of strawberry. *Galactomyces candidum* JYC1146 inhibited hyphal growth by 69.6 percent via volatile compound production and chitinase-mediated lysis of fungal cell walls, reducing gray mold incidence to 77.2 percent after 4 days and 47.7 percent after 8 days of storage(Chen et al. 2018). The yeast *Rhodotorula glutinis* suppressed both hyphal growth and spore germination of *Botrytis cinerea*, reducing disease incidence by over 94 percent after 2 days at 20 °C and 7 days at 4 °C (Zhang et al. 2007). This yeast colonized wound sites effectively and remained active under both ambient and cold conditions. The mycoparasitic fungus *Clonostachys rosea*, applied twice weekly under field conditions, decreased infection rates in flowers and fruit from 50.6 to 10.0 percent and from 25.1 to 5.9 percent, respectively, while reducing spore production and latent infection by more than 80 percent (Cota et al. 2008). *Bacillus halotolerans* KLBC XJ-5 decreased gray mold incidence from 100 to 6.7 percent and reduced lesion diameter by 25 percent. Its antagonistic effect was attributed to the production of antimicrobial compounds, secretion of chitinase, and induction of host resistance via increased PAL, PPO, chalcone isomerase, and β−1,3-glucanase activities (Wang et al. 2021).

Other fungal pathogens, including *Rhizopus stolonifer*, also cause substantial fruit decay during storage and handling. The yeast *Debaryomyces hansenii* has emerged as an effective biocontrol agent against soft rot caused by *Rhizopus stolonifer*. It reduced disease incidence from 81.5 to 14.8 percent by inhibiting spore germination and germ tube elongation, while simultaneously activating host defense enzymes such as SOD, POD, CAT, PPO, and PAL (Zhao et al. 2023, 2022). These enzymatic responses promoted the accumulation of phenolic and antioxidant compounds in fruit tissue, enhancing physiological resistance to pathogen invasion.

Collectively, these findings demonstrate that a wide range of microbial antagonists exert strong biocontrol activity against fruit pathogens in strawberry. Their effectiveness arises from multiple mechanisms, including the production of antifungal metabolites and VOCs, enzymatic degradation of pathogen cell walls, and the induction of host antioxidant and structural defenses that maintain fruit integrity and post-harvest quality.

## Future research directions for sustainable strawberry cultivation

The rise of multiple stresses imposed by a changing climate presents a challenge to the sustainability of strawberry production, despite its high plasticity, adaptability to different environments, and variety of cultivation methods. Cultivation under highly controlled greenhouse conditions, such as vertical farming, may increase resource use efficiency and minimize the impact of climate change on strawberry productivity and health (Verma et al. 2025). Such conditions require significant investment and have a high energy demand, however, thus fostering a rise in greenhouse gas emissions (Khoshnevisan et al. 2014). Such considerations limit the effectiveness of these technologies in addressing the food demands of a continuously growing global population. Thus, the development of sustainable methods of strawberry production relies on four complementary strategies. These, listed in their probable order of development from the short to the long term, are 1. microbial improvement of plant fitness and health; 2. novel cultural methods based on precision agriculture; 3. epigenetic manipulation; and 4. genetic improvement.

### Microbial improvement

**Fig. 4:**
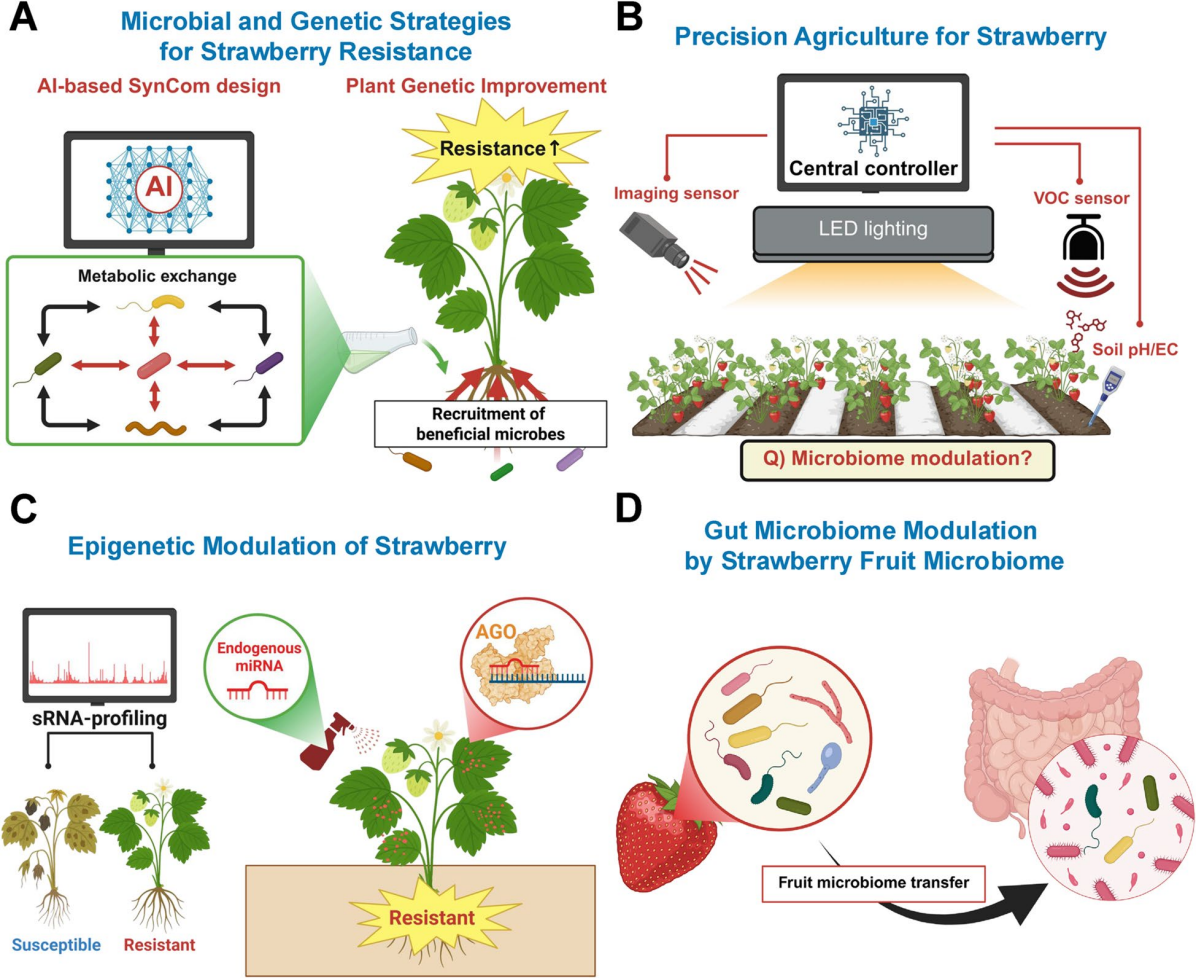
Integrated holobiome-based strategies with impact ranging from sustainable strawberry production to the human microbiome. This figure illustrates holobiome-based strategies for sustainable strawberry cultivation. It also shows the potential modulating influence of strawberry holobiomes on the human gut microbiome. **A** Microbial and genetic strategies for strawberry resistance. **B** Precision agriculture for strawberry cultivation. **C** Epigenetic modulation of strawberry. **D** Gut microbiome modulation by strawberry fruit microbiome

A promising approach to mitigating the impact of climate changes on strawberry productivity is the development of synthetic communities (SynComs) (Fig. 4A). SynCom development is still in its infancy and harnessing its full potential requires systematic and standardized studies (Berg et al. 2021). SynComs may be built by mixing selected strains and applying them to plants under controlled conditions (Mueller and Sachs 2015). Transcriptomic and proteomic analyses unveiled the mechanisms underlying plant–microbe interactions (Sergaki et al. 2018). SynComs introduce a second generation of microbiomic manipulation that reshapes the phytobiome composition to include desired traits. SynComs consist of multi-species consortia that include complementary modes of action, such as plant growth promotion, induction of resistance, and pathogen inhibition. The main challenge to the development of effective SynComs is maximizing facilitation over interspecific competition between the different species (Xu et al. 2025), which may occur after several cycles of SynCom inoculation onto plants (SynCom experimental evolution). One strategy for overcoming this problem is the use of Artificial Intelligence (AI) or Explainable Artificial Intelligence (XAI) to model microbial interaction within a SynCom and between SynCom members on the host plant (Jing et al. 2024). The XAI approach can unravel the causal links between microbes and plant immunity (Venneman et al. 2020), thus identifying the keystone taxa that contribute to plant health. Moreover, the combination of supervised machine learning models (Emmenegger et al. 2023) with explainable components will enable analysis of the relative importance of each taxon, thus defining the roles of microbes within a SynCom and generating new biomarkers. These models can be combined with metadata to recognize patterns using integrative multi-omics approache.

The design of SynComs applicable to strawberry is currently severely constrained by the paucity of high-resolution metagenomic datasets specific to strawberry-associated microbiomes. Most studies have focused on taxonomic profiling based on 16S rRNA or Internal Transcribed Spacer amplicon sequencing and provide limited functional insight and strain-level resolution. In such data-sparse environments, the identification of functionally relevant taxa, prediction of interspecies interactions, and modeling of ecological stability are highly speculative. This substantially hinders the development of customized, context-adapted SynComs capable of reliably shaping protective microbiomes in field-grown strawberry. Therefore, large-scale, function-oriented metagenomic and metatranscriptomic surveys targeting strawberry compartments and developmental stages are urgently required to enable precise and predictable SynCom engineering.

### New cultivation methods based on precision agriculture

Over the past decade, several complementary tools for sensing plant health and physiological status have been developed and integrated into Decision Support Systems (DSSs), enabling a precise application of different culture methods (Fig. 4B) (Lindblom et al. 2017). These tools include wearable chemosensors for detecting and monitoring VOCs (Li et al. 2021b), visible and near-infrared (visNIR), thermal, and hyperspectral imaging (Siedliska et al. 2018), multi-view remote imaging (Zheng et al. 2023), and environmental and soil sensors. Strawberry-specific data acquisition strategies have not yet been developed, however, especially for stationary fixed spectral analysis chambers and in-field remote sensing systems. Precision agriculture coupled with soil-less, indoor cultivation may create a complete closed-cycle production system (Hadavi and Ghazijahani 2018). The use of light-emitting diodes (LEDs) can increase the energy and resource use efficiency of such systems. Modulation of the light spectrum may increase crop yield and quality (Roosta et al. 2024) and influence plant-pathogen interactions (Correia et al. 2022), but the effects of these cultivation techniques and LED lighting on the assembly, structure, and functionality of the strawberry microbiome has not been yet fully elucidated.

### Epigenetic modulation of strawberry

RNA profiling (sRNA-omic and transcriptomic analyses) in susceptible *vs.* resistant strawberry cultivars is a novel approach allowing the development of sRNA through the identification of highly relevant sRNA effectors and their target genes (Koch and Wassenegger 2021). Understanding epigenetic changes in response to infection will identify differentially methylated genes. This is essential for epigenetics-based protection strategies that use approaches such as epigenetic editing (EpiEdit) and CRISPR-Cas9-modified EpiEdit systems. Once these tools are developed, RNA-directed host gene modulations may be achieved by spraying with non-coding (*e.g*., dsRNA, siRNA) or coding (*e.g.*, mRNA, circRNA) RNAs (Fig. 4C) (Höfle et al. 2020). In the future, such new control tools may be integrated into a precise application system based on plant sensors & XIA-generated DSS.

### Genetic improvement

The modern cultivated strawberry, *Fragaria* × *annanassa*, was generated less than 300 years ago by hybridizing *Fragaria virginiana* with *Fragaria chiloensis*. Strawberry resistance is mostly polygenic and quantitatively inherited, which makes it difficult to associate molecular markers with disease resistance genes against the main pathogens and/or abiotic stressors. The development of a new strawberry cultivar typically involves a lengthy breeding process and requires multiple years of hybridization and phenotypic selection. The accelerating pace of climate change thus creates a temporal mismatch with the timeframes needed for cultivar development. This misalignment significantly complicates the identification and selection of key agronomic and adaptive traits, thereby hindering the formulation of effective and climate-resilient breeding strategies. Furthermore, the octoploid genome of strawberry poses substantial challenges for the development and use of molecular markers for marker-assisted selection.

Microbial-driven breeding may help to overcome the problems facing traditional breeding programs (Fig. 4A). The “Plant Social Networking System” (pSNS) model (Sharifi and Ryu 2021; Park and Ryu 2021) suggests the existence of an unwired/wired plant–plant and plant–microbiome network, and also triggers/determinants to adjust defense responses to stressors. Crop plants may have lost some of their communication abilities during domestication and in breeding programs (Soldan et al. 2021). The development of novel tools to modulate plant traits to enable recruitment of beneficial microorganisms or to dissuade pathogens is thus a goal for strawberry breeding programs over the coming decades. Improving the strawberry microbiome via genetic modulation of the host plant must be achieved to enable plant-driven enrichment of beneficial microbial communities (Clouse and Wagner 2021).

### Impact of strawberry holobiomes on the human gut microbiome

Fruit and vegetables have long been recognized as sources of nutrients and bioactive compounds; however, they also serve as important reservoirs of diverse microbial communities. Despite this, the potential impact of plant-derived microbes on the human gut microbiome has long been overlooked. Microbes on fruits and vegetables can be transmitted to the human gut and persist there at detectable levels (Wicaksono et al. 2023). This study reconstructed 314 high-quality, non-redundant metagenome-assembled genomes (MAGs) from 156 fruit and vegetable metagenomes and mapped these to 2,426 human gut metagenomic datasets. Bacteria associated with fruit and vegetables were consistently present in the human gut microbiota, albeit at low relative abundance (0.003 to 3.6%). Microbial diversity and abundance increased over the weaning period (1–12 months of age), suggesting that plant-derived microbes were actively transferred to the gut during the early stages of solid food introduction.

Furthermore, the frequency and diversity of vegetable consumption was positively correlated with the detection rate and abundance of plant-associated MAGs, indicating that diet played a key role in mediating the integration of plant-derived microbes into the gut ecosystem. Notably, bacterial genera commonly found in strawberry fruit, such as *Pseudomonas*, *Sphingomonas*, and *Methylobacterium*, are also reported in the human gut, suggesting that strawberry-associated microbes influence gut microbial composition, either transiently or via colonization. The functional roles of these microbes in the gut remain poorly understood. These findings shift the paradigm from a purely nutrient-based view of food-microbiome interactions toward a new ecological perspective that considers the role of the food-borne microbes themselves (Fig. 4D).

## Conclusion

Strawberry is a popular fruit found on dining tables across the world. Strawberry production faces many new challenges, even as consumer requirements for quantity and quality increase sharply year on year. Climate change has had a major impact on strawberry cultivation, as on other crop plants. In addition, consumers and famers are increasing their demands for safe agriproducts because of the harmful side-effects of chemical control methods. Recent amplicon sequencing-based metagenomic analyses have advanced our understanding of the strawberry holobiome. Despite this, our knowledge of holobiome functionality and its application to agriculture remains at an early stage. We believe now is the right time to shift our viewpoint beyond single microbial preparations. In particular, strawberries should no longer be regarded solely as sources of nutrients and bioactive compounds, but also as ecologically meaningful microbial carriers. Future research is needed to determine whether strawberry-associated microbiota can colonize the human gut stably, modulate host physiology, and interact with the resident gut microbiota.

## Data Availability

Not applicable.

## Abbreviations

AMF: Arbuscular mycorrhizal fungi
CAT: Catalase
ISR: Induced systemic resistance
LOX: Lipoxygenase
PAL: Phenylalanine ammonia lyase
PGPR: Plant growth-promoting rhizobacteria
PKC1: Protein kinase C
POD: Peroxidase
PPO: Polyphenol oxidase
PR1: PR10: Pathogenesis-related proteins 1 and 10
RD19: Responsive to dehydration 19
RBOHD: Respiratory burst oxidase homolog D
SBE2: Suppressor of BEM4 protein 2
SOD: Superoxide dismutase
SynCom: Synthetic microbial community
VOC: Volatile organic compound
XAI: Explainable artificial intelligence

## References

[CR1] Agusti L, Bonaterra A, Moragrega C, Camps J, Montesinos E (2011) Biocontrol of root rot of strawberry caused by *Phytophthora cactorum* with a combination of two *Pseudomonas fluorescens* strains. J Plant Pathol 1:363–372

[CR2] Ahmed M, El-Fiki I (2017) Effect of biological control of root rot diseases of strawberry using *Trichoderma* spp. Middle East J Appl Sci 7(3):482–492

[CR3] Alijani Z, Amini J, Ashengroph M, Bahramnejad B, Mozafari AA (2022) Biocontrol of strawberry anthracnose disease caused by *Colletotrichum nymphaeae* using *Bacillus atrophaeus* strain DM6120 with multiple mechanisms. Trop Plant Pathol 47(2):245–259. 10.1007/s40858-021-00477-7

[CR4] Amidon KS (2008) Adolf Meyer-Abich, holism, and the negotiation of theoretical biology. Biol Theory 3:357–370. 10.1162/biot.2008.3.4.357

[CR5] Amrutha P, Vijayaraghavan R (2018) Evaluation of fungicides and biocontrol agents against *Neopestalotiopsis clavispora* causing leaf blight of strawberry (*Fragaria x ananassa* Duch.). Int J Curr Microbiol App Sci 7(8):622–628. 10.20546/ijcmas.2018.708.067

[CR6] Anandhakumar J, Zeller W (2004) Investigation on the Biocontrol of Phytophthora diseases on strawberry based on antagonism. Paper presented at the Ecofruit-11th International Conference on Cultivation Technique and Phytopathological Problems in Organic Fruit-Growing: Proceedings to the Conference, Weinsberg, Germany, 3-5 February 2004

[CR7] Bashir I, War AF, Rafiq I, Reshi ZA, Rashid I, Shouche YS (2022) Phyllosphere microbiome: diversity and functions. Microbiol Res 254:126888. 10.1016/j.micres.2021.126888 34700185 10.1016/j.micres.2021.126888

[CR8] Bass D, Stentiford GD, Wang H-C, Koskella B, Tyler CR (2019) The pathobiome in animal and plant diseases. Trends Ecol Evol 34(11):996–1008. 10.1016/j.tree.2019.07.012 31522755 10.1016/j.tree.2019.07.012 PMC7479508

[CR9] Berg G, Kusstatscher P, Abdelfattah A, Cernava T, Smalla K (2021) Microbiome modulation—toward a better understanding of plant microbiome response to microbial inoculants. Front Microbiol 2021:12. 10.3389/fmicb.2021.650610 10.3389/fmicb.2021.650610 PMC8060476 33897663

[CR10] Cha JY, Han S, Hong HJ, Cho H, Kim D, Kwon Y, Kwon SK, Crüsemann M, Bok Lee Y, Kim JF, Giaever G, Nislow C, Moore BS, Thomashow LS, Weller DM, Kwak YS (2015) Microbial and biochemical basis of a *Fusarium* wilt-suppressive soil. ISME J 10(1):119–129. 10.1038/ismej.2015.95 26057845 10.1038/ismej.2015.95 PMC4681868

[CR11] Chen P-H, Chen R-Y, Chou J-Y (2018) Screening and evaluation of yeast antagonists for biological control of *Botrytis cinerea* on strawberry fruits. Mycobiology 46(1):33–46. 10.1080/12298093.2018.1454013 29998031 10.1080/12298093.2018.1454013 PMC6037076

[CR12] Chen Y, Xu Y, Zhou T, Akkaya MS, Wang L, Li S, Li X (2020) Biocontrol of *Fusarium* wilt disease in strawberries using bioorganic fertilizer fortified with *Bacillus licheniformis* X-1 and *Bacillus methylotrophicus* Z-1. 3 Biotech 10(2):80. 10.1007/s13205-020-2060-6 32099731 10.1007/s13205-020-2060-6 PMC6992835

[CR13] Clouse KM, Wagner MR (2021) Plant genetics as a tool for manipulating crop microbiomes: opportunities and challenges. Front Bioeng Biotechnol 9:567548. 10.3389/fbioe.2021.567548 10.3389/fbioe.2021.567548 PMC8201784 34136470

[CR14] Compant S, Cassan F, Kostić T, Johnson L, Brader G, Trognitz F, Sessitsch A (2025) Harnessing the plant microbiome for sustainable crop production. Nat Rev Microbiol 23(1):9–23. 10.1038/s41579-024-01079-1 39147829 10.1038/s41579-024-01079-1

[CR15] Correia C, Magnani F, Pastore C, Cellini A, Donati I, Pennisi G, Paucek I, Orsini F, Vandelle E, Santos C, Spinelli F (2022) Red and blue light differently influence *Actinidia chinensis* performance and its interaction with *Pseudomonas syringae* pv. *Actinidiae*. Int J Mol Sci 23(21):13145. 10.3390/ijms232113145 36361938 10.3390/ijms232113145 PMC9658526

[CR16] Cota LV, Maffia LA, Mizubuti ESG, Macedo PEF, Antunes RF (2008) Biological control of strawberry gray mold by *Clonostachys rosea* under field conditions. Biol Control 46(3):515–522. 10.1016/j.biocontrol.2008.04.023

[CR17] Dara SK (2016) Managing strawberry pests with chemical pesticides and non-chemical alternatives. Int J Fruit Sci 16(sup1):129–141. 10.1080/15538362.2016.1195311

[CR18] Darrow GM (1966) The strawberry. History, breeding and physiology. Holt, Rinehart & Winston, New York

[CR19] De Cal A, Redondo C, Sztejnberg A, Melgarejo P (2008) Biocontrol of powdery mildew by *Penicillium oxalicum* in open-field nurseries of strawberries. Biol Control 47(1):103–107. 10.1016/j.biocontrol.2008.07.010

[CR20] De Tender C, Haegeman A, Vandecasteele B, Clement L, Cremelie P, Dawyndt P, Maes M, Debode J (2016) Dynamics in the strawberry rhizosphere microbiome in response to biochar and *Botrytis cinerea* leaf infection. Front Microbiol 7:2062. 10.3389/fmicb.2016.02062 28066380 10.3389/fmicb.2016.02062 PMC5177642

[CR21] Deng S, Wipf HML, Pierroz G, Raab TK, Khanna R, Coleman-Derr D (2019) A plant growth-promoting microbial soil amendment dynamically alters the strawberry root bacterial microbiome. Sci Rep 9(1):17677. 10.1038/s41598-019-53623-2 31776356 10.1038/s41598-019-53623-2 PMC6881409

[CR22] Dittmer J, Lusseau T, Foissac X, Faoro F (2021) Skipping the insect vector: plant stolon transmission of the phytopathogen ‘Ca. *Phlomobacter fragariae*’from the *Arsenophonus* clade of insect endosymbionts. Insects 12(2):93. 10.3390/insects12020093 33499057 10.3390/insects12020093 PMC7912703

[CR23] Duchesne A (1766) Histoire naturelle des fraisiers. Didot le jeune, Paris, France

[CR24] Emmenegger B, Massoni J, Pestalozzi CM, Bortfeld-Miller M, Maier BA, Vorholt JA (2023) Identifying microbiota community patterns important for plant protection using synthetic communities and machine learning. Nat Commun 14(1):7983. 10.1038/s41467-023-43793-z 38042924 10.1038/s41467-023-43793-z PMC10693592

[CR26] Fait A, Hanhineva K, Beleggia R, Dai N, Rogachev I, Nikiforova VJ, Fernie AR, Aharoni A (2008) Reconfiguration of the achene and receptacle metabolic networks during strawberry fruit development. Plant Physiol 148(2):730–750. 10.1104/pp.108.120691 18715960 10.1104/pp.108.120691 PMC2556830

[CR27] FAO (2023) Statistical databases. https://www.fao.org/statistics/en/

[CR28] Finn CE, Retamales JB, Lobos GA, Hancock JF (2013) The Chilean strawberry (*Fragaria chiloensis*): over 1000 years of domestication. HortScience 48(4):418–421. 10.21273/HORTSCI.48.4.418

[CR29] Giampieri F, Tulipani S, Alvarez-Suarez JM, Quiles JL, Mezzetti B, Battino M (2012) The strawberry: composition, nutritional quality, and impact on human health. Nutrition 28(1):9–19. 10.1016/j.nut.2011.08.009 22153122 10.1016/j.nut.2011.08.009

[CR30] Guerrero R, Margulis L, Berlanga M (2013) Symbiogenesis: the holobiont as a unit of evolution. Int Microbiol 16(3):133–143. 10.2436/20.1501.01.188 24568029 10.2436/20.1501.01.188

[CR31] Guerrero-Molina MF, Winik BC, Pedraza RO (2012) More than rhizosphere colonization of strawberry plants by *Azospirillum brasilense*. Appl Soil Ecol 61:205–212. 10.1016/j.apsoil.2011.10.011

[CR32] Hadavi E, Ghazijahani N (2018) Closed and Semi-closed Systems in Agriculture. In: Lichtfouse E (ed) Sustainable Agriculture Reviews 33: Climate Impact on Agriculture. Springer International Publishing, Cham, pp 295–310. 10.1007/978-3-319-99076-7_10

[CR33] Harel YM, Kolton M, Elad Y, Rav-David D, Cytryn E, Borenstein M, Shulchani R, Graber E (2011) Induced systemic resistance in strawberry (*Fragaria*× *ananassa*) to powdery mildew using various control agents. Paper presented at the the 6th IOBC meeting on Multitrophic Interactions in Soil, Cordoba, Spain, 4-7 April 2011

[CR34] Hassani MA, Gonzalez O, Hunter SS, Holmes GJ, Hewavitharana SS, Ivors K, Lazcano C (2023) Microbiome network connectivity and composition linked to disease resistance in strawberry plants. Phytobiomes J 7(3):298–311. 10.1094/pbiomes-10-22-0069-r

[CR35] Hernández-Martínez NR, Blanchard C, Wells D, Salazar-Gutiérrez MR (2023) Current state and future perspectives of commercial strawberry production: a review. Sci Hortic 312:111893. 10.1016/j.scienta.2023.111893

[CR36] Höfle L, Biedenkopf D, Werner BT, Shrestha A, Jelonek L, Koch A (2020) Study on the efficiency of dsRNAs with increasing length in RNA-based silencing of the Fusarium CYP51 genes. RNA Biol 17(4):463–473. 10.1080/15476286.2019.1700033 31814508 10.1080/15476286.2019.1700033 PMC7237133

[CR37] Hu X, Claerbout J, Vandecasteele B, Craeye S, Geelen D (2025) The bacterial and fungal strawberry root-associated microbiome in reused peat-based substrate. BMC Plant Biol 25(1):245. 10.1186/s12870-025-06217-2 39994558 10.1186/s12870-025-06217-2 PMC11849140

[CR38] Jamwal M, Sharma N (2019) Chapter 23 soilless culture. In: Sharma RM et al (eds) Strawberries: Production, Postharvest Management and Protection. 10.1201/b21441

[CR39] Jing J, Garbeva P, Raaijmakers JM, Medema MH (2024) Strategies for tailoring functional microbial synthetic communities. ISME J. 10.1093/ismejo/wrae049 38537571 10.1093/ismejo/wrae049 PMC11008692

[CR40] Khoshnevisan B, Shariati HM, Rafiee S, Mousazadeh H (2014) Comparison of energy consumption and GHG emissions of open field and greenhouse strawberry production. Renew Sustain Energy Rev 29:316–324. 10.1016/j.rser.2013.08.098

[CR41] Kim D-R, Cho G, Jeon C-W, Weller DM, Thomashow LS, Paulitz TC, Kwak Y-S (2019) A mutualistic interaction between *Streptomyces* bacteria, strawberry plants and pollinating bees. Nat Commun 10(1):4802. 10.1038/s41467-019-12785-3 31641114 10.1038/s41467-019-12785-3 PMC6805876

[CR42] Kim M-J, Shim C-K, Ko B-G, Kim J (2020) Effect of the microalga *Chlorella fusca* CHK0059 on strawberry PGPR and biological control of Fusarium wilt disease in non-pesticide hydroponic strawberry cultivation. J Microbiol Biotechnol 30(5):708. 10.4014/jmb.2001.01015 32482936 10.4014/jmb.2001.01015 PMC9728245

[CR43] Koch A, Wassenegger M (2021) Host-induced gene silencing – mechanisms and applications. New Phytol 231(1):54–59. 10.1111/nph.17364 33774815 10.1111/nph.17364

[CR44] Kukkurainen S, Leino A, Vahamiko S, Karkkainen H, Ahanen K, Sorvari S, Rugienius R, Toldi O (2005) Occurrence and location of endophytic bacteria in garden and wild strawberry. HortScience 40(2):348–352. 10.21273/HORTSCI.40.2.348

[CR45] Kurze S, Bahl H, Dahl R, Berg G (2001) Biological control of fungal strawberry diseases by *Serratia plymuthica* HRO-C48. Plant Dis 85(5):529–534. 10.1094/pdis.2001.85.5.529 30823130 10.1094/PDIS.2001.85.5.529

[CR46] Labadie M, Guy K, Demené M-N, Caraglio Y, Heidsieck G, Gaston A, Rothan C, Guédon Y, Pradal C, Denoyes B (2023) Spatio-temporal analysis of strawberry architecture: insights into the control of branching and inflorescence complexity. J Exp Bot 74(12):3595–3612. 10.1093/jxb/erad097 37133320 10.1093/jxb/erad097 PMC10299788

[CR47] Lazcano C, Boyd E, Holmes G, Hewavitharana S, Pasulka A, Ivors K (2021) The rhizosphere microbiome plays a role in the resistance to soil-borne pathogens and nutrient uptake of strawberry cultivars under field conditions. Sci Rep 11(1):3188. 10.1038/s41598-021-82768-2 33542451 10.1038/s41598-021-82768-2 PMC7862632

[CR48] Lee B-Y, Chen P-L, Chen C-Y (2024) Suppression of strawberry anthracnose by *Paenibacillus polymyxa* TP3 in situ and from a distance. Plant Dis 108(3):700–710. 10.1094/pdis-08-23-1499-re 37580883 10.1094/PDIS-08-23-1499-RE

[CR49] Legein M, Smets W, Wuyts K, Bosmans L, Samson R, Lebeer S (2022) The greenhouse phyllosphere microbiome and associations with introduced bumblebees and predatory mites. Microbiol Spectr 10(4):e01755-01722. 10.1128/spectrum.01755-22 10.1128/spectrum.01755-22 PMC9431046 35862945

[CR50] Li W-h, Liu Q-z (2019) Changes in fungal community and diversity in strawberry rhizosphere soil after 12 years in the greenhouse. J Integr Agric 18(3):677–687. 10.1016/S2095-3119(18)62003-9

[CR51] Li X, Jing T, Zhou D, Zhang M, Qi D, Zang X, Zhao Y, Li K, Tang W, Chen Y (2021a) Biocontrol efficacy and possible mechanism of *Streptomyces* sp. H4 against postharvest anthracnose caused by *Colletotrichum fragariae* on strawberry fruit. Postharvest Biol Technol 175:111401. 10.1016/j.postharvbio.2020.111401

[CR52] Li Z, Liu Y, Hossain O, Paul R, Yao S, Wu S, Ristaino JB, Zhu Y, Wei Q (2021b) Real-time monitoring of plant stresses via chemiresistive profiling of leaf volatiles by a wearable sensor. Matter 4(7):2553–2570. 10.1016/j.matt.2021.06.009

[CR53] Li X, Zhang L, Zhao Y, Feng J, Chen Y, Li K, Zhang M, Qi D, Zhou D, Wei Y (2024) Biocontrol potential of volatile organic compounds produced by *Streptomyces corchorusii* CG-G2 to strawberry anthracnose caused by *Colletotrichum gloeosporioides*. Food Chem 437:137938. 10.1016/j.foodchem.2023.137938 37948803 10.1016/j.foodchem.2023.137938

[CR54] Lindblom J, Lundström C, Ljung M, Jonsson A (2017) Promoting sustainable intensification in precision agriculture: review of decision support systems development and strategies. Precis Agric 18(3):309–331. 10.1007/s11119-016-9491-4

[CR55] Liston A, Cronn R, Ashman TL (2014) *Fragaria*: a genus with deep historical roots and ripe for evolutionary and ecological insights. Am J Bot 101(10):1686–1699. 10.3732/ajb.1400140 25326614 10.3732/ajb.1400140

[CR56] Marco S, Loredana M, Riccardo V, Raffaella B, Walter C, Luca N (2022) Microbe-assisted crop improvement: a sustainable weapon to restore holobiont functionality and resilience. Hortic Res. 10.1093/hr/uhac160 36204199 10.1093/hr/uhac160 PMC9531342

[CR57] Margulis L, Fester R (eds) (1991) Symbiosis as a source of evolutionary innovation: speciation and morphogenesis. MIT press, Cambridge, Mass. and London 11538111

[CR58] Mendes R, Kruijt M, De Bruijn I, Dekkers E, Van Der Voort M, Schneider JH, Piceno YM, DeSantis TZ, Andersen GL, Bakker PA (2011) Deciphering the rhizosphere microbiome for disease-suppressive bacteria. Science 332(6033):1097–1100. 10.1126/science.1203980 21551032 10.1126/science.1203980

[CR59] Mochizuki M, Yamamoto S, Aoki Y, Suzuki S (2012) Isolation and characterisation of *Bacillus amyloliquefaciens* S13–3 as a biological control agent for anthracnose caused by *Colletotrichum gloeosporioides*. Biocontrol Sci Technol 22(6):697–709. 10.1080/09583157.2012.679644

[CR60] Mueller UG, Sachs JL (2015) Engineering microbiomes to improve plant and animal health. Trends Microbiol 23(10):606–617. 10.1016/j.tim.2015.07.009 26422463 10.1016/j.tim.2015.07.009

[CR61] Nallanchakravarthula S, Mahmood S, Alström S, Finlay RD (2014) Influence of soil type, cultivar and *Verticillium dahliae* on the structure of the root and rhizosphere soil fungal microbiome of strawberry. PLoS One 9(10):e111455. 10.1371/journal.pone.0111455 25347069 10.1371/journal.pone.0111455 PMC4210224

[CR62] Nam M-H, Park M-S, Kim H-G, Yoo S-J (2009) Biological control of strawberry Fusarium wilt caused by *Fusarium oxysporum* f. sp. *fragariae* using *Bacillus velezensis* BS87 and RK1 formulation. J Microbiol Biotechnol 19(5):520–524 19494701 10.4014/jmb.0805.333

[CR63] Nam JH, Thibodeau A, Qian YL, Qian MC, Park SH (2023) Multidisciplinary evaluation of plant growth promoting rhizobacteria on soil microbiome and strawberry quality. AMB Express 13(1):18. 10.1186/s13568-023-01524-z 36795258 10.1186/s13568-023-01524-z PMC9935790

[CR64] Oğuz İ, İbrahim Oğuz H, Ebru Kafkas N (2022) Strawberry cultivation techniques. In: Recent Studies on Strawberries. IntechOpen. 10.5772/intechopen.104611

[CR65] Olimi E, Kusstatscher P, Wicaksono WA, Abdelfattah A, Cernava T, Berg G (2022) Insights into the microbiome assembly during different growth stages and storage of strawberry plants. Environ Microbiol 17(1):21. 10.1186/s40793-022-00415-3 10.1186/s40793-022-00415-3 PMC9052558 35484554

[CR66] Park Y-S, Ryu C-M (2021) Understanding plant social networking system: avoiding deleterious microbiota but calling beneficials. Int J Mol Sci 22(7):3319. 10.3390/ijms22073319 33805032 10.3390/ijms22073319 PMC8037233

[CR67] Pastrana A, Watson D, Gordon T (2019) Transmission of *Fusarium oxysporum* f. sp. *fragariae* through stolons in strawberry plants. Plant Dis 103(6):1249–1251. 10.1094/pdis-08-18-1353-re 30932736 10.1094/PDIS-08-18-1353-RE

[CR68] Pastrana AM, Basallote-Ureba MJ, Aguado A, Akdi K, Capote N (2016) Biological control of strawberry soil-borne pathogens Macrophomina phaseolina and Fusarium solani, using Trichoderma asperellum and Bacillus spp. Phytopathol Mediterra 55:109–120. 10.14601/PHYTOPATHOL_MEDITERR-16363

[CR69] Pertot I, Zasso R, Amsalem L, Baldessari M, Angeli G, Elad Y (2008) Integrating biocontrol agents in strawberry powdery mildew control strategies in high tunnel growing systems. Crop Prot 27(3):622–631. 10.1016/j.cropro.2007.09.004

[CR70] Porras M, Barrau C, Arroyo FT, Santos B, Blanco C, Romero F (2007) Reduction of *Phytophthora cactorum* in Strawberry Fields by *Trichoderma* spp. and soil solarization. Plant Dis 91(2):142–146. 10.1094/PDIS-91-2-0142 30780995 10.1094/PDIS-91-2-0142

[CR71] Priyadarshi R, Jayakumar A, de Souza CK, Rhim JW, Kim JT (2024) Advances in strawberry postharvest preservation and packaging: a comprehensive review. Compr Rev Food Sci Food Saf 23(4):e13417. 10.1111/1541-4337.13417 39072989 10.1111/1541-4337.13417

[CR72] Raza MM, Bebber DP (2022) Climate change and plant pathogens. Curr Opin Microbiol 70:102233. 10.1016/j.mib.2022.102233 36370642 10.1016/j.mib.2022.102233

[CR73] Roberts P, Berger R, Jones J, Chandler C, Stall R (1997) Disease progress, yield loss, and control of *Xanthomonas fragariae* on strawberry plants. Plant Dis 81(8):917–921. 10.1094/pdis.1997.81.8.917 30866381 10.1094/PDIS.1997.81.8.917

[CR74] Roosta HR, Bikdeloo M, Ghorbanpour M (2024) The growth, nutrient uptake and fruit quality in four strawberry cultivars under different spectra of LED supplemental light. BMC Plant Biol 24(1):179. 10.1186/s12870-024-04880-5 38454341 10.1186/s12870-024-04880-5 PMC10921718

[CR75] Rudrappa T, Czymmek KJ, Paré PW, Bais HP (2008) Root-secreted malic acid recruits beneficial soil bacteria. Plant Physiol 148(3):1547–1556. 10.1104/pp.108.127613 18820082 10.1104/pp.108.127613 PMC2577262

[CR76] Sakamoto M, Uenishi M, Miyamoto K, Suzuki T (2016) Effect of root-zone temperature on the growth and fruit quality of hydroponically grown strawberry plants. J Agric Sci 8(5):122–131. 10.5539/jas.v8n5p122

[CR77] Sangiorgio D, Cellini A, Donati I, Ferrari E, Tanunchai B, Fareed Mohamed Wahdan S, Sadubsarn D, Farneti B, Checcucci A, Buscot F, Spinelli F, Purahong W (2022a) Taxonomical and functional composition of strawberry microbiome is genotype-dependent. J Adv Res 42:189–204. 10.1016/j.jare.2022.02.009 36513413 10.1016/j.jare.2022.02.009 PMC9788945

[CR78] Sangiorgio D, Spinelli F, Vandelle E (2022b) The unseen effect of pesticides: the impact on phytobiota structure and functions. Front Agron 4:936032. 10.3389/fagro.2022.936032

[CR79] Sayyed RZ, Ilyas N (eds) (2024) Plant holobiome engineering for climate-smart agriculture. Springer, Singapore. 10.1007/978-981-99-9388-8

[CR80] Schneider L, Rebetez M, Rasmann S (2022) The effect of climate change on invasive crop pests across biomes. Curr Opin Insect Sci 50:100895. 10.1016/j.cois.2022.100895 35240333 10.1016/j.cois.2022.100895

[CR81] Sergaki C, Lagunas B, Lidbury I, Gifford ML, Schäfer P (2018) Challenges and approaches in microbiome research: from fundamental to applied. Front Plant Sci 2018:9. 10.3389/fpls.2018.01205 10.3389/fpls.2018.01205 PMC6107787 30174681

[CR82] Sharifi R, Ryu C-M (2021) Social networking in crop plants: wired and wireless cross-plant communications. Plant Cell Environ 44(4):1095–1110. 10.1111/pce.13966 33274469 10.1111/pce.13966 PMC8049059

[CR83] Siedliska A, Baranowski P, Zubik M, Mazurek W, Sosnowska B (2018) Detection of fungal infections in strawberry fruit by VNIR/SWIR hyperspectral imaging. Postharvest Biol Technol 139:115–126. 10.1016/j.postharvbio.2018.01.018

[CR84] Simpson D (2018) The Economic Importance of Strawberry Crops. In: Hytönen T, Graham J, Harrison R (eds) The Genomes of Rosaceous Berries and Their Wild Relatives. Springer International Publishing, Cham, pp 1–7. 10.1007/978-3-319-76020-9_1

[CR85] Sohrabi R, Paasch BC, Liber JA, He SY (2023) Phyllosphere microbiome. Annu Rev Plant Biol 74:539–568. 10.1146/annurev-arplant-102820-032704 36854478 10.1146/annurev-arplant-102820-032704

[CR86] Soldan R, Fusi M, Cardinale M, Daffonchio D, Preston GM (2021) The effect of plant domestication on host control of the microbiota. Commun Biol 4(1):936. 10.1038/s42003-021-02467-6 34354230 10.1038/s42003-021-02467-6 PMC8342519

[CR87] Song L, Zhong Z, Han Y, Zheng Q, Qin Y, Wu Q, He X, Pan C (2020) Dissipation of sixteen pesticide residues from various applications of commercial formulations on strawberry and their risk assessment under greenhouse conditions. Ecotoxicol Environ Saf 188:109842. 10.1016/j.ecoenv.2019.109842 31707322 10.1016/j.ecoenv.2019.109842

[CR88] Sowik I, Borkowska B, Markiewicz M (2016) The activity of mycorrhizal symbiosis in suppressing *Verticillium* wilt in susceptible and tolerant strawberry (*Fragaria x ananassa* Duch.) genotypes. Appl Soil Ecol 101:152–164. 10.1016/j.apsoil.2016.01.021

[CR89] Stone BW, Weingarten EA, Jackson CR (2018) The role of the phyllosphere microbiome in plant health and function. Ann Plant Rev Online:533–556. 10.1002/9781119312994.apr0614

[CR90] Su D, Chen S, Zhou W, Yang J, Luo Z, Zhang Z, Tian Y, Dong Q, Shen X, Wei S (2022) Comparative analysis of the microbial community structures between healthy and anthracnose-infected strawberry rhizosphere soils using Illumina sequencing technology in Yunnan Province, Southwest of China. Front Microbiol 13:881450. 10.3389/fmicb.2022.881450 35651487 10.3389/fmicb.2022.881450 PMC9149601

[CR91] Thapa S, Prasanna R (2018) Prospecting the characteristics and significance of the phyllosphere microbiome. Ann Microbiol 68(5):229–245. 10.1007/s13213-018-1331-5

[CR92] Todeschini V, AitLahmidi N, Mazzucco E, Marsano F, Gosetti F, Robotti E, Bona E, Massa N, Bonneau L, Marengo E, Wipf D, Berta G, Lingua G (2018) Impact of beneficial microorganisms on strawberry growth, fruit production, nutritional quality, and volatilome. Front Plant Sci. 10.3389/fpls.2018.01611 30505312 10.3389/fpls.2018.01611 PMC6250784

[CR93] Trivedi P, Leach JE, Tringe SG, Sa T, Singh BK (2020) Plant–microbiome interactions: from community assembly to plant health. Nat Rev Microbiol 18(11):607–621. 10.1038/s41579-020-0412-1 32788714 10.1038/s41579-020-0412-1

[CR94] Trnka M, Brázdil R, Olesen JE, Eitzinger J, Zahradníček P, Kocmánková E, Dobrovolný P, Štěpánek P, Možný M, Bartošová L (2012) Could the changes in regional crop yields be a pointer of climatic change? Agric For Meteorol 166:62–71. 10.1016/j.agrformet.2012.05.020

[CR95] Tuomainen TV, Toljamo A, Kokko H, Nissi MJ (2024) Non-invasive assessment and visualization of *Phytophthora cactorum* infection in strawberry crowns using quantitative magnetic resonance imaging. Sci Rep 14(1):2129. 10.1038/s41598-024-52520-7 38267614 10.1038/s41598-024-52520-7 PMC10808117

[CR96] Vandenkoornhuyse P, Quaiser A, Duhamel M, Le Van A, Dufresne A (2015) The importance of the microbiome of the plant holobiont. New Phytol 206(4):1196–1206. 10.1111/nph.13312 25655016 10.1111/nph.13312

[CR97] Venneman J, Vandermeersch L, Walgraeve C, Audenaert K, Ameye M, Verwaeren J, Steppe K, Van Langenhove H, Haesaert G, Vereecke D (2020) Respiratory CO2 combined with a blend of volatiles emitted by endophytic *Serendipita* strains strongly stimulate growth of *Arabidopsis* implicating auxin and cytokinin signaling. Front Plant Sci. 10.3389/fpls.2020.544435 32983211 10.3389/fpls.2020.544435 PMC7492573

[CR98] Verma P, Singh G, Singh SK, Mirza AA, Bakshi M, Anmol, Lakshya, Kumar L, Rupesh (2025) Modulating Productivity of Strawberries (Fragaria x ananassa Duch.) through artificial full-spectrum light in indoor vertical farming. Journal of Soil Science and Plant Nutrition 25 (1):1219-1233. 10.1007/s42729-024-02197-8

[CR99] Viejobueno J, Albornoz PL, Camacho M, de los Santos B, Martínez-Zamora MG, Salazar SM (2021a) Protection of strawberry plants against charcoal rot disease (*Macrophomina phaseolina*) induced by *Azospirillum brasilense*. Agronomy 11(2):195. 10.3390/agronomy11020195

[CR100] Viejobueno J, Rodríguez-Berbel N, Miranda L, de los Santos B, Camacho M (2021b) Potential bacterial antagonists for the control of charcoal rot (*Macrophomina phaseolina*) in strawberry. Horticulturae 7(11):457. 10.3390/horticulturae7110457

[CR101] Voges MJ, Bai Y, Schulze-Lefert P, Sattely ES (2019) Plant-derived coumarins shape the composition of an Arabidopsis synthetic root microbiome. Proc Natl Acad Sci U S A 116(25):12558–12565. 10.1073/pnas.1820691116 31152139 10.1073/pnas.1820691116 PMC6589675

[CR102] Wang D, Geng Z, Shen J, Xie T, Wu F, Zhang Y, Dong Y, Mao D, Ji Y, Huang Y (2025) Biocontrol effects of volatile organic compounds released from *Burkholderia sola* NAU20 to strawberry anthracnose caused by *Colletotrichum gloeosporioides*. Postharvest Biol Technol 225:113510. 10.1016/j.postharvbio.2025.113510

[CR103] Wang F, Xiao J, Zhang Y, Li R, Liu L, Deng J (2021) Biocontrol ability and action mechanism of *Bacillus halotolerans* against *Botrytis cinerea* causing grey mould in postharvest strawberry fruit. Postharvest Biol Technol 174:111456. 10.1016/j.postharvbio.2020.111456

[CR104] Wardlaw J, Davis C, MacLeod C, Robertson A (2023) Pesticide Usage in Scotland, Soft Fruit Crops 2022. Scottish Government, Edinburgh

[CR105] Wei Z, Jousset A (2017) Plant breeding goes microbial. Trends Plant Sci 22(7):555–558. 10.1016/j.tplants.2017.05.009 28592368 10.1016/j.tplants.2017.05.009

[CR106] Wicaksono WA, Cernava T, Wassermann B, Abdelfattah A, Soto-Giron MJ, Toledo GV, Virtanen SM, Knip M, Hyöty H, Berg G (2023) The edible plant microbiome: evidence for the occurrence of fruit and vegetable bacteria in the human gut. Gut Microbes 15(2):2258565. 10.1080/19490976.2023.2258565 37741805 10.1080/19490976.2023.2258565 PMC10519362

[CR107] Winter CK, Katz JM (2011) Dietary exposure to pesticide residues from commodities alleged to contain the highest contamination levels. J Toxicol 2011(1):589674. 10.1155/2011/589674 21776262 10.1155/2011/589674 PMC3135239

[CR108] Wu Y-M, Chen X, Wang F, Hsiao C-Y, Yang C-Y, Lin S-T, Wu L-H, Chen Y-K, Liang Y-S, Lin Y-H (2021) *Bacillus amyloliquefaciens* strains control strawberry anthracnose through antagonistic activity and plant immune response intensification. Biol Control 157:104592. 10.1016/j.biocontrol.2021.104592

[CR109] Xu X, Dinesen C, Pioppi A, Kovács ÁT, Lozano-Andrade CN (2025) Composing a microbial symphony: synthetic communities for promoting plant growth. Trends Microbiol 33(7):738–751. 10.1016/j.tim.2025.01.006 39966007 10.1016/j.tim.2025.01.006

[CR110] Yanan W, Xusheng Z, Baozhong Y, Wenchao Z, Jintang G (2015) Biochemical defenses induced by mycorrhizae fungi *Glomus mosseae* in controlling strawberry *Fusarium* wilt. Open Biomed Eng J 9:301–304. 10.2174/1874120701509010301 26998177 10.2174/1874120701509010301 PMC4774392

[CR111] Yang H, Zhang J, Zhang H, Cao R, Tang D, Wang L (2024a) 5-aminolevulinic acid against strawberry *Fusarium* wilt: bidirectional regulation of biocontrol agents and pathogens. Hortic Plant J 10(6):1349–1361. 10.1016/j.hpj.2023.02.014

[CR112] Yang W, Wang M, Wang H, Zhang C, Zhang Q, Xiao H (2024b) Exploitation of the biocontrol potential of a marine-derived *Bacillus velezensis* and its application on postharvest strawberry. Food Control 161:110311. 10.1016/j.foodcont.2024.110311

[CR113] Yang H, Zhang X, Yan Z, Wang Y, Wang Q, Lu B, Chen J, Wu X (2025) Diversity and function of strawberry endophytic bacterial communities associated with host genotype and niche. Curr Microbiol 82(6):244. 10.1007/s00284-025-04223-z 40237826 10.1007/s00284-025-04223-z PMC12003465

[CR114] Zhang H, Wang L, Dong Y, Jiang S, Cao J, Meng R (2007) Postharvest biological control of gray mold decay of strawberry with *Rhodotorula glutinis*. Biol Control 40(2):287–292. 10.1016/j.biocontrol.2006.10.008

[CR115] Zhang J, Wang X, Yu O, Tang J, Gu X, Wan X, Fang C (2010) Metabolic profiling of strawberry (*Fragaria*×*ananassa* Duch.) during fruit development and maturation. J Exp Bot 62(3):1103–1118. 10.1093/jxb/erq343 21041374 10.1093/jxb/erq343

[CR116] Zhang M, Kong Z, Fu H, Shu X, Xue Q, Lai H, Guo Q (2023a) Rhizosphere microbial ecological characteristics of strawberry root rot. Front Microbiol 14:1286740. 10.3389/fmicb.2023.1286740 10.3389/fmicb.2023.1286740 PMC10687216 38033596

[CR117] Zhang S, Wu J, Chen J, Jun S, Yuan Y, Dai X, Wang F, Ma Y (2024) The biological control effect of *Bacillus cereus* on strawberry leaf spot disease caused by *Neopestalotiopsis clavispora*. Sci Hortic 327:112841. 10.1016/j.scienta.2024.112841

[CR118] Zhang X, Ling C, Wu X, Fan S, Liang Q, Zhou F (2023b) Bacterial diversity and function shift of strawberry root in different cultivation substrates. Rhizosphere 26:100696. 10.1016/j.rhisph.2023.100696

[CR119] Zhang Y, Fan T, Jia W, Zhang W, Liu Q, Li B, Zhang L (2012) Identification and characterization of a *Bacillus subtilis* strain TS06 as bio-control agent of strawberry replant disease (*Fusarium* and *Verticilium* wilts). Afr J Biotechnol 11(3):570–580. 10.5897/AJB11.1131

[CR120] Zhao L, Lan C, Tang X, Li B, Zhang X, Gu X, Zhang H (2022) Efficacy of *Debaryomyce hansenii* in the biocontrol for postharvest soft rot of strawberry and investigation of the physiological mechanisms involved. Biol Control 174:105011. 10.1016/j.biocontrol.2022.105011

[CR121] Zhao L, Zhou Y, Liang L, Godana EA, Zhang X, Yang X, Wu M, Song Y, Zhang H (2023) Changes in quality and microbiome composition of strawberry fruits following postharvest application of *Debaryomyces hansenii*, a yeast biocontrol agent. Postharvest Biol Technol 202:112379. 10.1016/j.postharvbio.2023.112379

[CR122] Zheng C, Liu T, Abd-Elrahman A, Whitaker VM, Wilkinson B (2023) Object-detection from multi-view remote sensing images: a case study of fruit and flower detection and counting on a central Florida strawberry farm. Int J Appl Earth Obs Geoinf 123:103457. 10.1016/j.jag.2023.103457

[CR123] Zhimo VY, Kumar A, Biasi A, Salim S, Feygenberg O, Toamy MA, Abdelfattaah A, Medina S, Freilich S, Wisniewski M, Droby S (2021) Compositional shifts in the strawberry fruit microbiome in response to near-harvest application of *Metschnikowia fructicola*, a yeast biocontrol agent. Postharvest Biol Technol 175:111469. 10.1016/j.postharvbio.2021.111469

